# Adaptive Divergence without Distinct Species Relationships Indicate Early Stage Ecological Speciation in Species of the *Rhododendron* *pseudochrysanthum* Complex Endemic to Taiwan

**DOI:** 10.3390/plants11091226

**Published:** 2022-04-30

**Authors:** Jia-Jia Cao, Yi-Shao Li, Chung-Te Chang, Jeng-Der Chung, Shih-Ying Hwang

**Affiliations:** 1 Department of Life Science, National Taiwan University, 1 Roosevelt Road, Section 4, Taipei 10617, Taiwan; r05b21034@ntu.edu.tw; 2 School of Life Science, National Taiwan Normal University, 88 Tingchow Road, Section 4, Taipei 11677, Taiwan; yishaoli@ntnu.edu.tw; 3 Department of Life Science, Tunghai University, 1727 Taiwan Boulevard, Section 4, Taichung 40704, Taiwan; changchuante@gmail.com; 4 Division of Silviculture, Taiwan Forestry Research Institute, 53 Nanhai Road, Taipei 10066, Taiwan; chung@tfri.gov.tw

**Keywords:** ecological speciation, environmentally-associated dependent evolution, genetic and epigenetic variation, incomplete lineage sorting, local adaptation

## Abstract

The testing association of environmental variables with genetic and epigenetic variation could be crucial to deciphering the effects of environmental factors playing roles as selective drivers in ecological speciation. Although ecological speciation may occur in closely related species, species boundaries may not be established over a short evolutionary timescale. Here, we investigated the genetic and epigenetic variations using amplified fragment length polymorphism (AFLP) and methylation-sensitive amplification polymorphism (MSAP), respectively, and tested their associations with environmental variables in populations of four closely related species in the *R. pseudochrysanthum* complex. No distinctive species relationships were found using genetic clustering analyses, neighbor-joining tree, and neighbor-net tree based on the total AFLP variation, which is suggestive of the incomplete lineage sorting of ancestral variation. Nonetheless, strong isolation-by-environment and adaptive divergence were revealed, despite the significant isolation-by-distance. Annual mean temperature, elevation, normalized difference vegetation index, and annual total potential evapotranspiration were found to be the most important environmental variables explaining outlier genetic and epigenetic variations. Our results suggest that the four closely related species of the *R. pseudochrysanthum* complex share the polymorphism of their ancestor, but reproductive isolation due to ecological speciation can occur if local environmental divergence persists over time.

## 1. Introduction

Divergence between species and between populations in close association with environments is an important aspect of research in evolutionary biology [1]. Local ecological lineages are expected to be invoked by environmental heterogeneity [2] and may result in ecological speciation [3]. Range shifts via expansions toward polar and high elevation range limits are expected and the dispersal capability is crucial for species adaptation and survival [4,5]. A biogeographical upward shift of distribution in response to postglacial climatic warming is commonly found [6,7]. Populations shifting upward may evoke adaptive divergence at elevational marginal populations [8,9,10], albeit leading-edge populations are likely under survival threat when facing with habitat reduction and high-elevation environments [7,11]. Trailing-edge populations may be the most vulnerable and may be under the risk of extinction if global change dramatically exceeds a species’ past rates of thermal niche evolution [9,10,12]. Nonetheless, locally adapted alleles that correspond to environmental conditions can be found in trailing- and leading-edge populations at the species’ range margins [9,10,11,12].

Natural selection driven by environmental factors can act on both genetic and epigenetic variations, leading to ecological adaptation and speciation [10,13,14,15,16]. Researchers can quantify genetic variation in natural populations by using techniques involving next generation sequencing (NGS), such as restriction site-associated sequencing [17]. Amplified fragment length polymorphism (AFLP) [18], despite being less powerful than NGS techniques, is an efficient technique which reveals hundreds of molecular markers generated from the genome sequences of non-model organisms that may be involved in adaptive evolution [19,20]. Epigenetic variation can influence gene expression without DNA sequence alteration [15,16]. Genome-wide changes in methylation status can be accessed via methods, such as the genome-wide sequencing of cytosine methylation [21], which are dependent on detailed genome sequence information. In non-model organisms, epigenetic variation can be quantified using methylation-sensitive amplification polymorphism (MSAP) [22], which is independent of the availability of the genome sequence information. Stably inherited epigenetic variation may play a crucial role at the interface between the environment and the genome [15,16,23]. Epialleles occurring in natural populations associated strongly with environment can be important for population adaptive evolution and survival [23,24].

The *Rhododendron pseudochrysanthum* complex comprises four closely related species, including *R. hyperythrum, R. morii, R. pseudochrysanthum*, and *R. rubropunctatum*, that belong to the subgenus *Hymenanthes* [25]. At elevations above 3000 m, *R. pseudochrysanthum* grows on the periphery of cold adapted coniferous forests. *R. morii* inhabits the periphery of warm-temperate evergreen broadleaved forests at elevations around 2400–3000 m in central and southern Taiwan. Populations of these two species overlap at elevations of approximately 3000 m in the mountains of Hohuanshan and Tahsueshan in central Taiwan [25]. *R. hyperythrum* is found dominating the alpine tundra at an elevation of around 3500 m on the peak of Nanhutashan in central Taiwan. *R. rubropunctatum* is distributed in the northern subtropical evergreen broadleaved forests at elevations around 600–1200 m [25].

Close phylogenetic relationships were observed between the species of the *R. pseudochrysanthum* complex based on chloroplast DNA (cpDNA) sequences [26]. Populations of this species complex experienced north-to-south expansions during the last glacial maximum (LGM) [26]. Effective population size reductions of high-elevation Hohuanshan and Tahsueshan populations were found [10], based on the expressed sequence tag simple sequence repeats (EST-SSRs) data, indicating past range retractions because of the upward range shifts to higher elevations [5,6,7]. The current distributions of species in the *R. pseudochrysanthum* complex are the outcome of the past upward migration of *R. hyperythrum, R. morii,* and *R. pseudochrysanthum* populations to high elevations and the restriction of *R. rubropunctatum* populations in northern lower elevations. Only one out of the 26 EST-SSR loci assessed was found to be an environmentally-dependent selective outlier in global comparison and was also in pair population comparisons involving the low-elevation *R. rubropunctatum* populations [10]. Although the chance of potential advantageous mutations may be reduced if the effective population size decreases [1,2,3,4,5,13], population adaptive divergence found in species with low effective population size is not uncommon, particularly when selection is strong [2,3,13,14].

Tree species may diverge ecologically and lead to reproductive isolation because of divergent environments [1,2,3]. Selection can create a pattern of isolation-by-environment (IBE) [27,28] in contrast to gene flow between populations impeded solely by geographic distance (isolation-by-distance, IBD). Closely related species can be in various stages of divergence, before complete reproductive isolation is invoked by environmental differences [1]. The detection of adaptive divergence in the elevational trailing- and leading-edge populations is probable in the *R. pseudochrysanthum* complex, due to its demographic history of glacial expansion and postglacial upward shifting, using a large number of molecular markers. Here, 171 and 132 samples from nine and six populations of the *R. pseudochrysanthum* complex were surveyed for AFLP and MSAP variation, respectively. The genetic and epigenetic variations surveyed were used to test the hypothesis of the early stage ecological speciation in the *R. pseudochrysanthum* complex.

The AFLP and MSAP variations surveyed were used in a combination of phylogenetic; genetic clustering; genetic differentiation; genome scans; and multivariate analytic techniques to answer four specific questions: (1) Is there species integrity in the four closely related species of the *R. pseudochrysanthum* complex? (2) Are AFLP and MSAP *F*_ST_ outliers; detected by genome scans methods; associated strongly with environmental variables? (3) Does IBE play a stronger role than IBD in explaining outlier genetic and epigenetic variations? And (4) What are the most important environmental variables contributing to outlier genetic and epigenetic variations? Answering these questions will provide information to investigate the probable evolutionary process shaping the patterns of genetic diversity and phylogenetic relationships and a probability of ecological speciation of four closely related species in the *R. pseudochrysanthum* complex.

## 2. Results

### 2.1. Genetic Diversity Based on the Total AFLP Variation

**Figure 1:**
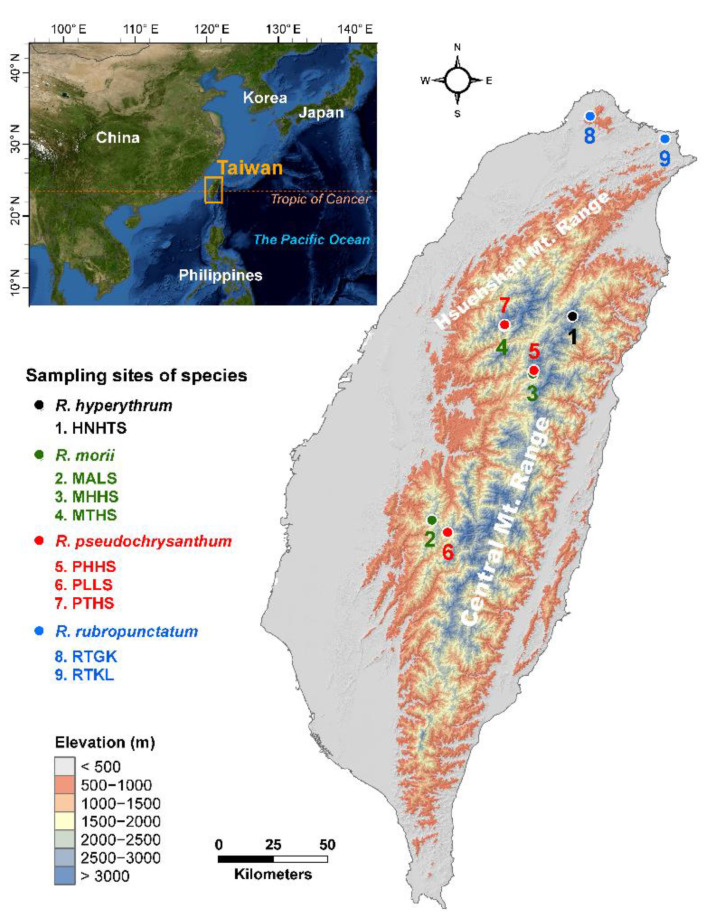
Geographic distribution of the nine populations of four closely related species in the *Rhododendron pseudochrysanthum* complex occurring in Taiwan. The countries’ boundary (polygon) map was derived from the default map database in ArcGIS v.10.3 (Supplementary Methods). The elevation gradients of Taiwan (background) were presented in ArcGIS based on the 20 m digital elevation model. The locations of the sampling sites were plotted using Tools in ArcGIS by their coordinates. See Table 1 for abbreviations of the nine populations of the *R. pseudochrysanthum* complex.

With 171 individuals of the *R. pseudochrysanthum* complex (Table 1, Figure 1), eight primer combinations generated a total of 384 AFLP loci with an overall repeatability of 96.2% (Table S1). The proportion of polymorphic loci ranged from 56.0% (population PTHS) to 69.8% (population PLLS) with an average of 62.0% (Table 1). The average level of unbiased expected heterozygosity (*uH*_E_) was 0.2211 and ranged from 0.2038 (population PTHS) to 0.2439 (population RTGK). The analysis with the linear mixed effect model (LMM) showed no overall *uH*_E_ significant difference when compared between species (Wald *χ*^2^ = 4.4342, *p* = 0.2182). Only the population pair between PLLS and PTHS had a significant *uH*_E_ difference after Tukey’s *p* value adjustment (*t* = −2.637, *p* = 0.0085; Table S2), albeit an overall significant difference was revealed (Wald *χ*^2^ = 21.241, *p* = 0.0065). The test for multilocus linkage disequilibrium revealed a significant departure from random associations in both the index of association (*I*_A_) and the modified index of association (*r*_D_) between AFLP loci (Table 1).

### 2.2. Environmental Heterogeneity

Overall environmental heterogeneity between the sampling sites was found using permutational multivariate analysis of variance (PERMANOVA), based on the 11 retained environmental variables (Supplementary Methods, Table S3) (*p* = 0.001). These environmental variables include: bioclimatic (BIO1, annual mean temperature; BIO2, mean of the difference of the monthly maximum and minimum temperatures; and BIO12, annual precipitation); topographic (aspect, elevation, and slope); and ecological (CLO, cloud cover; NDVI, normalized difference vegetation index; PET, annual total potential evapotranspiration; RH, relative humidity; and WSmean, mean wind speed) variables. When compared between sample sites of different species, either significant or non-significant environmental differences can be found (Table S4). However, no significant environmental difference was found when comparing between the sample sites of the same species.

### 2.3. Genetic Relationships and Clustering Based on the Total AFLP Variation

**Figure 2:**
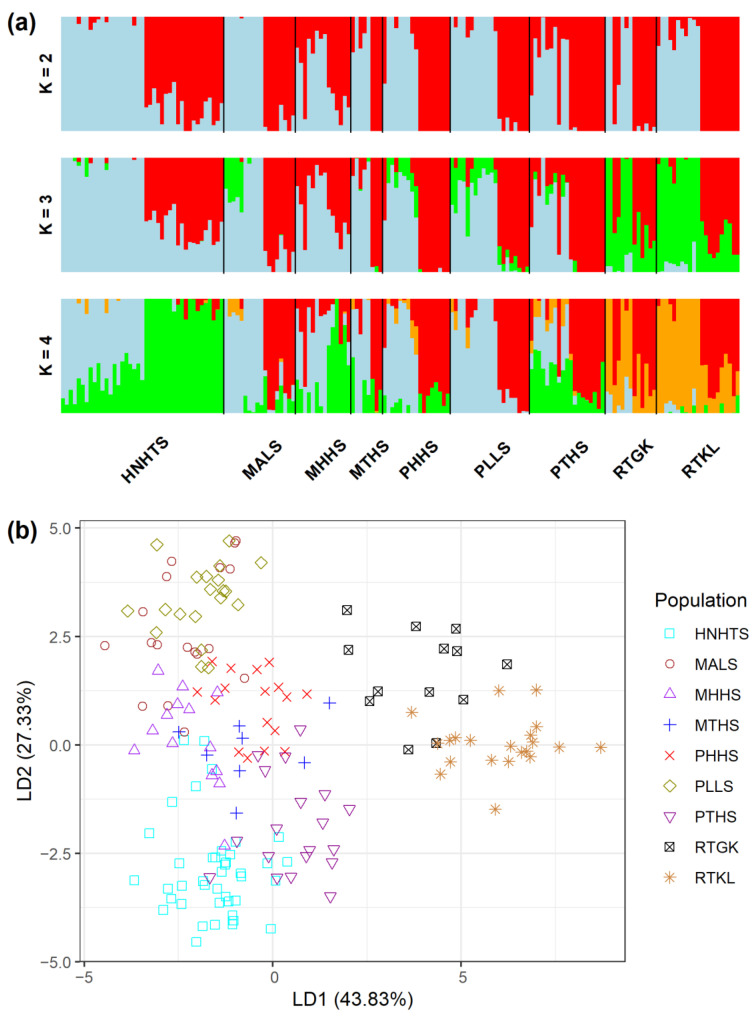
Analysis of genetic homogeneous groups of 171 individuals from nine populations of four closely related species in the *Rhododendron pseudochrysanthum* complex based on the total AFLP variation using (**a**) LEA and (**b**) DAPC. The clustering scenarios for *K* = 2–4 were displayed in LEA. LEA, landscape and ecological association [29]; DAPC, discriminant analysis of principal component [30,31].

**Figure 3:**
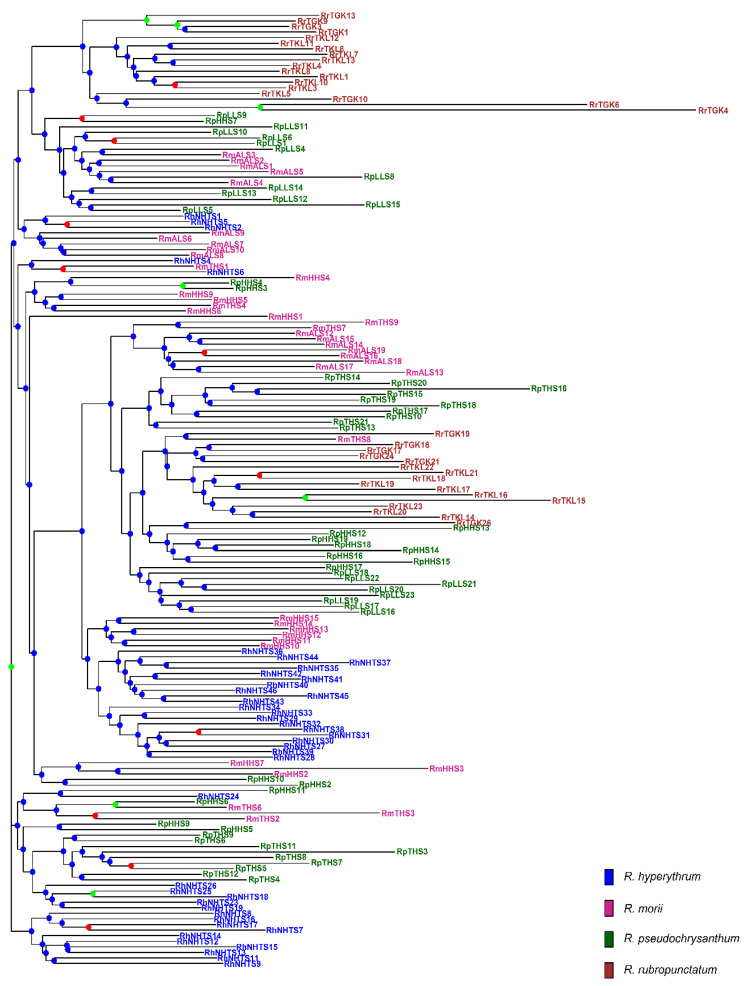
The neighbor-joining (NJ) tree of 171 individuals of four closely related species in the *Rhododendron pseudochrysanthum* complex based on the total AFLP variation. The NJ tree was generated based on Nei’s genetic distances [33] and 1000 bootstrap replicates were used in calculating bootstrap support values. Tip labels for individuals are colored: *R. hyperythrum* (blue), *R. morii* (violet red), *R. pseudochrysanthum* (dark green), and *R. rubropunctatum* (brown). For each node, bootstrap support values greater than 70%, between 50% and 70%, and smaller than 50% were coded with green, red, and blue circles, respectively.

The analysis of molecular variance (AMOVA) revealed shallow, but significant species differentiation (*Φ*_CT_ = 0.0520, *p* = 0.012; Table 2) based on the total AFLP data. The level of genetic differentiation between populations within species was significant (*Φ*_SC_ = 0.1000, *p* = 0.001). A significantly moderate level of differentiation between populations of the *R. pseudochrysanthum* complex was found (*Φ*_ST_ = 0.1468, *p* = 0.001; *F*_ST_ = 0.0763, *p* < 0.001). Moderate levels of genetic differentiation (*F*_ST_) were also found to be significant for all pairwise population comparisons (Table S5). Using the total AFLP data, the mean of the minimal cross entropy (CE) was minimized at *K* = 6 (Figure S1a) and the lowest Bayesian information criterion (BIC) was found at *K* = 5 (Figure S1b), respectively, using the sNMF algorithm of landscape and ecological association (LEA) [29] and the discriminant analysis of principal component (DAPC) [30,31]. Distinct population classification cannot be found, due to the high degree of shared polymorphism between individuals of different populations, as was revealed by LEA (Figure 2a). Using DAPC, three population clusters can be distinguished (Figure 2b). DAPC cluster 1 contains the two low-elevation populations RTGK and RTKL of *R. rubropunctatum*. DAPC cluster 2 contains individuals of populations MALS, PHHS, and PLLS, which belong to *R. morii* and *R. pseudochrysanthum*. DAPC cluster 3 included individuals of populations HNHTS, MTHS, and PTHS, which belong to *R. hyperythrum*, *R. morii*, and *R. pseudochrysanthum*. Nonetheless, individuals of populations HNHTS, PHHS, PTHS, and PLLS agglomerated in the periphery of clusters 2 and 3. The individual neighbor-joining (NJ) tree, generated based on the total AFLP data, with mostly low bootstrap values, revealed no distinctive relationships of individuals between populations and between species (Figure 3). Additionally, the center of the neighbor-net (NN) tree [32] was deeply intertwined and netted, which is supportive of no clear population and species distinction in the *R. pseudochrysanthum* complex (Figure S2). These results indicate the intermingling of individuals between populations of different species located in different geographic areas.

### 2.4. Potential Genetic and Epigenetic Outliers Associated with Environmental Variables and the Most Important Environmental Variables Explaining Outlier Variation

Out of 384 AFLP, 580 MSAP-m (methylated), and 274 MSAP-u (unmethylated) loci (Supplementary Methods, Table S6), 16 (4.2%), 4 (0.7%), and 15 (5.5%) loci, respectively, showed evidence of being *F*_ST_ outliers for population differentiation using both BAYESCAN [34] and DFDIST [35] in global comparisons (). The AFLP and MSAP primer pairs for amplification of these 35 outliers and the amplified length (bp) were listed in Table S7. These 35 loci were found to be associated with various environmental variables using LFMM (Latent factor mixed model) [36], Samβada [37], and a Bayesian logistic regression (*brm*) [38,39]. High levels of genetic differentiation at different hierarchical structures were found with *Φ*_CT_ = 0.2283 (*p* = 0.011), *Φ*_SC_ = 0.3099 (*p* = 0.001), *Φ*_ST_ = 0.4675 (*p* = 0.001) (Table 2), and among population *F*_ST_ = 0.2929 (*p* < 0.001), based on the variation of the 16 outlier AFLP loci.

**Figure 4:**
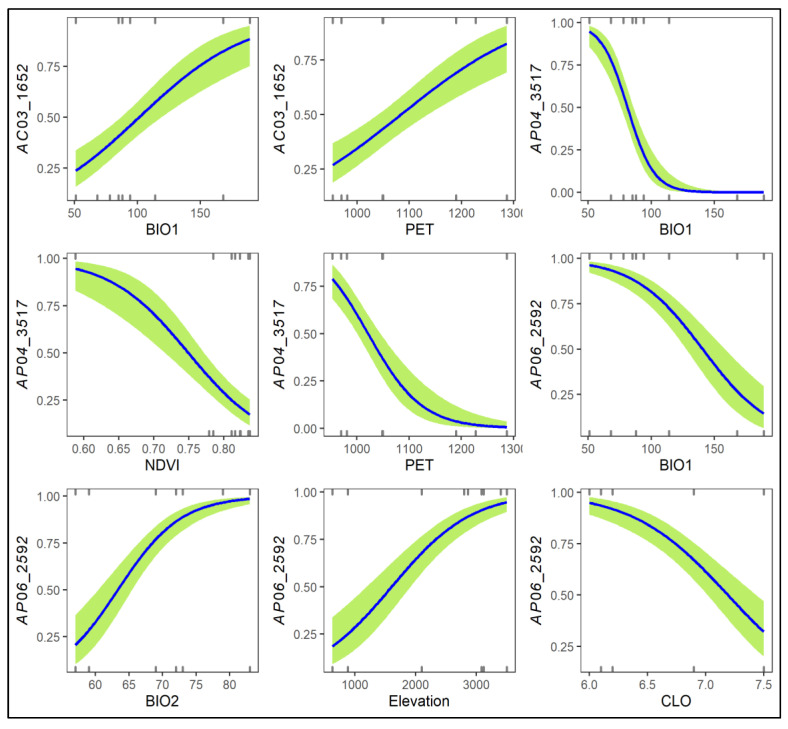
Logistic regression plots of the three AFLP loci associated strongly with environmental variables when comparing the low-elevation *Rhododendron rubropunctatum* populations with the high-elevation populations of species including *R. hyperythrum*, *R. morii*, and *R. pseudochrysanthum*. Values of the *y*-axis are the predicted probabilities of AFLP loci and the number of the *x*-axis are the values of environmental variables. Logistic regression was performed based on the generalized linear model with a logit link function and a binomial residual distribution. The presence/absence of the three loci (AC03_1652, AP04_3517, and AP06_2592) were used as response variables and the environmental variables strongly correlated with the loci were used as predictor variables in analysis using the *glm* function of R (Supplementary Methods). BIO1, annual mean temperature; BIO2, mean of the difference of the monthly maximum and minimum temperatures; CLO, cloud cover; NDVI, normalized difference vegetation index, PET, annual total potential evapotranspiration.

Three AFLP loci, including AC03_1652, AP04_3517, and AP06_2592, were detected as *F*_ST_ outliers and associated with environmental variables when compared between low-elevation trailing-edge *R. rubropunctatum* populations with high-elevation leading-edge populations of *R. hyperythrum*, *R. morii*, and *R. pseudochrysanthum* (Table S6). AC03_1652 was the potential selective outlier when compared between the RTKL population of *R. rubropunctatum* (high allele frequency) and populations MHHS and MTHS of *R. morii* (low allele frequencies) (Table S6, Figure S3). AP04_3517 was the selective outlier in comparison of the *R. hyperythrum* HNHTS population (high allele frequency) with both *R. rubropunctatum* populations (RTGK and RTKL; low allele frequencies). AP06_2592 was found to be the selective outlier when comparing *R. pseudochrysanthum* populations PHHS and PLLS (high allele frequencies) to the *R. rubropunctatum* RTGK population (low allele frequency). AC03_1652 was strongly correlated with BIO1 and PET. AP04_3517 was significantly correlated with BIO1, NDVI, and PET. AP06_2592 was found to be strongly correlated with BIO1, BIO2, elevation, and CLO. The probability estimates of these three AFLP outlier loci against the associated environmental gradients were depicted in Figure 4. Low allele frequencies in low-elevation trailing-edge populations in contrast to high allele frequencies in high-elevation leading-edge populations were found for AFLP outliers such as AC05_1828, AC05_2733, and AP06_3422 (Table S6, Figure S3). However, these loci were not detected as *F*_ST_ outliers by either DFDIST or BAYESCAN in pair population comparisons (Table S6).

No apparent contrasting differences in allele frequencies comparing the trailing- and leading-edge populations were found in all 15 outlier MSAP-u loci. Although a higher MSAP-m MP5_1240 allele frequency in the low-elevation trailing-edge population (RTKL) was found in contrast to high-elevation leading-edge populations with lower allele frequencies, no MSAP locus was detected as *F*_ST_ outlier by either BAYESCAN or DFDIST when comparing low-elevation trailing-edge populations to high-elevation leading-edge populations (Table S6).

Eleven environmental variables, including three bioclimatic (BIO1, BIO2, and BIO12), three topographic (aspect, elevation, and slope), and five ecological (CLO, NDVI, PET, RH, and WSmean) variables (Table S3), were used separately for forward selection analysis [40] to assess their contributions in explaining outlier AFLP variation. For the investigation of the contributions of environmental variables in explaining outlier MSAP variation, ecological variables CLO and WSmean were removed due to collinearity with the other three ecological variables in the six populations examined for MSAP. The most important environmental variables in the three environmental categories were BIO1 (adjusted *R*^2^ = 0.1576), elevation (adjusted *R*^2^ = 0.1394), and PET (adjusted *R*^2^ = 0.1150), respectively, explaining the outlier AFLP variation (Table 3). The most important environmental variables in the three environmental categories were BIO1 (adjusted *R*^2^ = 0.1925), elevation (adjusted *R*^2^ = 0.1272), and PET (adjusted *R*^2^ = 0.1664), respectively, explaining the outlier MSAP-m variation. BIO1 (adjusted *R*^2^ = 0.3446), elevation (adjusted *R*^2^ = 0.1772), and NDVI (adjusted *R*^2^ = 0.3518) were the most important environmental variables in the three environmental categories, respectively, explaining the outlier MSAP-u variation.

### 2.5. Relative Contribution of IBD and IBE Explaining Outlier Genetic and Epigenetic Variations

The retained 11 and 9 environmental variables were used in testing for IBD and IBE, respectively, based on the total variation. Significant relationships between environmental and geographic distances were found using a Mantel test and multiple matrix randomization regression (MMRR) [41] (Table 4). Except for the MSAP-m data, significant IBD was found in all the analyses based on the total variation using a Mantel test and MMRR. Partial Mantel tests found significant IBE based on the total AFLP and MSAP-u variations. A significant adaptive divergence can be inferred based on the three outlier datasets, controlling for geography using the partial Mantel test (AFLP: Mantel *r* = 0.2858, *p* = 0.001; MSAP-m: Mantel *r* = 0.1551, *p* = 0.001; MSAP-u: Mantel *r* = 0.0825, *p* = 0.002).

MMRR implements a combined model of geographic and environmental distances, which revealed patterns of significant IBE based on the total data. MMRR showed that environmental and geographic factors together explained 19.02% AFLP, 0.46% MSAP-m, and 5.09% MSAP-u variation of the total data; and 28.97% AFLP, 10.74% MSAP-m, and 4.93% MSAP-u variation of the outlier data. Additionally, MMRR revealed genetic and epigenetic adaptive divergences strongly correlated with environments based on the outlier datasets (AFLP: *β*_E_= 0.3074, *p* = 0.001 vs. *β*_D_ = 0.2488, *p* = 0.001; MSAP-m: *β*_E_ = 0.3412, *p* = 0.001 vs. *β*_D_ = −0.0175, *p* = 0.5755; MSAP-u: *β*_E_ = 0.3431, *p* = 0.001 vs. *β*_D_ = −0.1351, *p* = 0.001; Table 4).

## 3. Discussion

Genetic diversity in natural plant populations can be shaped by the mating system, pollen and seed dispersal, life form, past incidents such as range expansions, bottlenecks or founder events, and natural selection [7,10,20,26]. The levels of AFLP diversity were also found to be lower in the four species of the *R. pseudochrysanthum* complex compared with other *Rhododendron* species, including *R. calophytum*, *R. purdomii*, *R. concinnum*, *R. clementinae,* and *R. capitatum* [42]. However, the levels of AFLP diversity in the species of the *R. pseudochrysanthum* complex were similar to a narrowly distributed endangered species, *Rhododendron protistum* var. *giganteum* [43]. The species level AFLP diversity in the *R. pseudochrysanthum* complex was also similar to the average AFLP diversity summarized for 13 plant species [19]. Maintaining intraspecific genetic diversity is critical for a species to adapt and crucial to short-term and long-term survival [2,3,44]. Outcrossing is the predominant mating system in *Rhododendron* [45,46,47] and is thought to enhance the level of population genetic diversity; however, the relatively lower levels of population genetic diversity in species of the *R. pseudochrysanthum* complex might have resulted from past evolutionary history, such as population retractions due to postglacial upward shifting [10]. Since the LGM, a 1500 to 1600 m upward migration of forests was reported [48] and the population sizes of tree species, such as *Rhododendron*, in Taiwan are expected to decrease [7,10]. Range reductions can have significant genetic and evolutionary impacts, resulting in the loss of genetic diversity and consequences for population survival, and detrimental effects may have a marked influence on the distribution of marginal populations [49,50].

Although gene flow plays a critical role in shaping the current population genetic structure, the degree of gene flow estimated empirically may also reflect the population demographic history [51]. The DAPC clustering results demonstrated the apparent distinction of individuals of *R. rubropunctatum* from individuals of other species in the *R. pseudochrysanthum* complex (Figure 2b), but such inference cannot be made based on the results of LEA (Figure 2a), the NJ tree (Figure 3), and the NN tree (Figure S2). These results suggest that the four closely related species in the *R. pseudochrysanthum* complex can be combined into a single species [52] and are consistent with the results obtained based on cpDNA [26] and nuclear internal transcribed spacer sequences [53].

The pattern of relatively homogeneous levels of genetic diversity (Table S2) can be expected under an incomplete lineage sorting scenario [54]. The omnipresence of the intermingling of individuals between populations and between species, indicated by both NJ and NN trees (Figure 3, Figure S2), suggests short divergence times between taxa with historical large population sizes and/or the retention of ancestral polymorphism [10,26]. Nonetheless, these results may also be caused by the hybridization within the *R. pseudochrysanthum* complex, in which individuals of different populations and different species are grouped into the same clades. The predominant insect pollination in *Rhododendron* [55,56] and the tiny, winged seeds produced are likely to be dispersed by wind over a distance of approximately 30–80 m [56,57]; long distance recurrent gene flow leading to hybridization among individuals of different populations of the *R. pseudochrysanthum* complex is less likely. However, historical migration cannot be excluded [10,26]. The hypothesis predicts a positive correlation between the pairwise population spatial distance and population genetic differentiation due to limited gene flow, and historical gene flow can be inferred by regressing the pairwise population differentiation on geographic distance [54]. In the present study, historical gene flow can be inferred because of the significant correlations of pairwise *F*_ST_ (estimated based on the total AFLP) with Euclidean distances between the sample sites, calculated based on geographic coordinates, which were were found using Spearman’s rank correlation test (*ρ* = 0.669, S = 2570, *p* < 0.0001; Figure S4).

There are more than 200 peaks exceeding 3000 m in elevation with varied geographic topographies in mountainous regions in Taiwan. Because gravity is a crucial factor in seed dispersal [47,56], the contemporary seed dispersal of *Rhododendron* might be even more limited, considering that elevational differences and mountain ranges can be effective barriers to genetic exchange [58] in the distribution range of this species complex. Moreover, the general significant IBD pattern suggests a dispersal limitation at the spatial scale, which was assessed using a Mantel test and MMRR (Table 4). However, the current gene flow between species distributed in close proximity is probable, particularly in the case of *R. pseudochrysanthum* and *R. morii,* because their flowering times overlap, with the later flowers during March to May and the former flowers during April to June. Overlapping flowering times can result in a low level of genetic differentiation such as between population MHHS and population PTHS (*F*_ST_ = 0.0660), which are distributed in Hohuanshan (Table S5).

Environmental differences due to landscape heterogeneity in various deep valleys and high peaks of mountainous regions in Taiwan may play a role in limiting rather than promoting *Rhododendron* dispersal, and IBE would be relatively pronounced in contrast to IBD. Strong IBE indicates habitat isolation or immigrant inviability and may lead to reproductive isolation due to local environmental conditions, resulting in the reduced survival and reproduction of migrants [27,28]. We combined the use of one measure of environmental distance matrix generated from multiple environmental variables and an individual-based approach [59]; strong adaptive divergence was found, using a partial Mantel test and MMRR regression analysis, based on the outlier datasets (Table 4). Because geography and environment are not mutually exclusive in influencing genetic variation, both IBD and IBE can be effective in restricting gene flow between populations via direct and indirect processes [17,20,27,28,41]. Our results showed strong adaptive divergence using both the partial Mantel test and MMRR based on the outlier AFLP and MSAP datasets, suggesting that adaptive evolution caused by environmental differences may be important to the on-going and future process of ecological speciation in the *R. pseudochrysanthum* complex.

Three outlier loci (AC03_1652, AP04_3517, and AP06_2592) were found by pairwise comparisons between the leading- and trailing-edge populations, with high or low allele frequencies, indicating an adaptation associated with local environments. Increases in the levels of genetic divergence and the rate of speciation are, among others, found to be closely associated with temperature [60,61]. Temperature shifts have been found to play prominent roles in driving adaptive genetic and epigenetic variations in various plant species [10,17,20,23,24,62,63]. Our results suggest that temperature was the most important bioclimatic factor, with a high adjusted *R*^2^ value (Table 3), explaining the genetic and epigenetic variations among populations of the *R. pseudochrysanthum* complex. PET is a measure that accounts for water loss via transpiration [64] and was found to be the most important ecological factor (Table 3) highly associated with adaptive AFLP and MSAP-m variations (Table S6). PET was found to be associated with adaptive genetic variation in *Picea glauca* [65] and may be related to the increase in the adaptive capacity of trees under global warming in a drying climate. PET-related drought stress has also been found to be associated with epigenetic variation in *Vicia faba* [66]. Genetic differences between *Saccharum* species [67] and between populations of *Populus angustifolia* [68] were found to be related to the difference in traits that have the ability to maintain a favorable water balance. NDVI is a measure of surface coverage activity, representing the degree of vegetation greenness, suggestive of a biotic competitive environment that might play a role in interactions with other species in a local ecological community [69,70]. NDVI has been shown to be correlated with epigenetic variation in a coniferous species, *Taiwania cryptomerioides* [71]. The measurement of NDVI can range from −1 to 1, and a higher NDVI value indicates greater plant health [72]. The habitat of *R. hyperythrum* had a relatively lower NDVI value compared with that of other populations of the *R. pseudochrysanthum* complex (Table S3). The *R. hyperythrum* population at a high elevation might have evolved a local adaptation because of a selective outlier (AP04_3517) (Table S6) with a very high allele frequency in contrast to the extremely low allele frequencies of low-elevation populations (Figure S3). However, the habitat of the *R. hyperythrum* population with a low NDVI value suggests that it may be under threat of high elevation environments (e.g., a high UV condition).

Elevation is the most important topographic factor explaining outlier genetic and epigenetic variations (Table 3). Environmental variables can be classified into those highly correlated with altitude, such as annual mean temperature (*r* = −0.973), and those with a lower correlation coefficient, such as PET (*r* = −0.668) and NDVI (*r* = −0.420) in this study [73], which could have played roles in driving the population adaptive genetic and epigenetic divergences in the *R. pseudochrysanthum* complex. The elevational difference in meters is not a factor driving population divergence and may not be a useful predictor for distribution modeling [74]. However, elevation-dependent environmental conditions can be complex [75] and altitude-associated abiotic conditions may play important roles in shaping non-random variations of outlier allele frequencies and resulting in spatially-structured intraspecific genetic and epigenetic variations [10,24,76,77]. It is interesting that we found a higher probability estimate of an outlier AFLP locus (AP06_2592) with high allele frequencies at high-elevation populations (Figure 4). This locus was found to be significantly positively or negatively correlated with the environmental variables examined, such as annual mean temperature, mean of the difference of the monthly maximum and minimum temperatures, and cloud cover (Figure 4, Table S6, Figure S3). Additionally, elevational differences explaining outlier genetic and epigenetic variations may not only represent those abiotic factors examined in this study but also those that are not examined [78].

## 4. Materials and Methods

### 4.1. Sampling, Genotyping, and Epigenotyping

We collected fresh leaf samples of 171 individuals from nine populations of the *R. pseudochrysanthum* complex (Table 1, Figure 1) and used them for total DNA extraction [79] (Supplementary Methods). We surveyed the genetic variation of 171 individuals from nine populations using AFLP (Table 1). Due to a technical problem, 132 individuals from six populations were used in epigenotyping using MSAP. In AFLP, a total of 10 µL reaction volume, containing 6 µL (200 ng) of total genomic DNA digested with 0.5 µL *Eco*RI (20 UµL^−1^) and mixed with 1 µL *Mse*I (10 UµL^−1^), 1.5 µL ddH_2_O, and 1 µL CutSmart buffer (New England Biolabs, Ipswich, MA, USA), was incubated at 37 °C for 1.5 h. The reaction was terminated at 65 °C for 15 min. The digested DNA products were ligated with 1 µL (5 µM) of the *Eco*RI adaptor and 1 µL (50 µM) of the *Mse*I adaptor using 1 µL (5 UµL^−1^) T4 DNA ligase (Thermo Scientific, Vilnius, Lithuania), 3 µL ddH_2_O, and 4 µL 5X ligation buffer (Thermo Scientific, Vilnius, Lithuania) in a 10 µL ligation reaction mixture at 22 °C for 1 h.

Pre-selective amplification was performed using 4 µL diluted digested samples (1:9 dilution with ddH_2_O) as a template in a 20 µL volume containing 8.6 µL ddH_2_O, 2 µL 10X PCR buffer (Zymeset Biotech, Taipei, Taiwan), 1.6 µL *Eco*RI (16 µM; E00: 5′-GACTGCGTACCAATTC-3′), 1.6 µL *Mse*I (16 µM; M00: 5′-GATGAGTCCTGAGTAA-3′) primers, 1.6 µL dNTPs (2.5 mM), 0.4 µL MgCl_2_ (0.15 mM), and 0.2 µL *Taq* DNA polymerase (5 UµL^−1^; Zymeset). The pre-selective amplification was performed with an initial holding at 72 °C for 2 min and pre-denaturation at 94 °C for 3 min, followed by 25 cycles of 30 s at 94 °C, 30 s at 56 °C, and 1 min at 72 °C, with a final 5 min holding at 72 °C. Eight *Eco*RI-*Mse*I (E00 and M00) selective primer combinations with additional bases added at the ends were used for AFLP selective amplification (Table S1). *Eco*RI selective primer was labeled with fluorescent dye (6-carboxyfluorescein or hexachloro-fluorescein) and amplification was performed in a 20 µL volume containing 11.3 µL ddH_2_O, 2 µL 10X PCR buffer (Zymeset), 2 µL *Eco*RI (20 µM), 2 µL *Mse*I (20 µM) primers, 1.6 µL dNTPs (2.5 mM), 0.1 µL *Taq* DNA polymerase (5 UµL^−1^; Zymeset), and 1 µL diluted pre-selective amplified product (1:19 dilution with ddH_2_O). We performed selective amplification with an initial holding at 94°C for 3 min, followed by 13 cycles of 30 s at 94 °C, 30 s at 65–56 °C (decreasing the temperature by 0.7 °C each cycle), 1 min at 72 °C, then 23 cycles of 30 s at 94 °C, 30 s at 56 °C, and 1 min at 72 °C, with a final 5 min holding at 72 °C. In MSAP, the AFLP protocol was adapted by replacing restriction enzyme *Mse*I with the methylation-sensitive enzymes *Hpa*II and *Msp*I in two separate experiments. Ten MSAP selective primer combinations were used with additional nucleotides at the ends of the E00 and HM00 (*Hpa*II-*Msp*I, 5′-ATCATGAGTCCTGCTCGG-3′) (Table S1).

PCR amplification products were electrophoresed on an ABI 3730XL DNA analyzer and scored with Peak Scanner v.1.0 (Applied Biosystem, Foster City, CA, USA). We scored AFLP and MSAP fragments using a fluorescent threshold set at 150 units in the range of 100–500 bp. The “mixed scoring 1” of the *MSAP-calc* R script [80] in the R environment [81] was used to transform MSAP markers to two distinct types of data: MSAP-m (methylated) and MSAP-u (unmethylated) datasets [82]. Error rate per locus for AFLP and MSAP were calculated (Supplementary Methods, Table S1). Loci with an error rate per locus greater than 5% were removed [83].

### 4.2. Genetic Diversity Based on the Total AFLP Variation

The proportion of polymorphic loci and *uH*_E_ [84,85] were estimated using AFLP-SURV v.1.0 [86]. *uH*_E_ per locus was estimated using ARLEQUIN v.6.0 [87]. To test departure from linkage equilibrium indicating a possibility of inbreeding or non-random associations between alleles, measures of multilocus linkage disequilibrium, including *I*_A_ [88] and *r*_D_ [89], were estimated using the *ia* function of R poppr package [90] with 999 permutations. LMM, considering population as a fixed factor and locus as a random factor, was used to test the difference of mean *uH*_E_ per locus among species and among populations using the *lmer* function of R lme4 package [91], and significance was tested using the *Anova* function of R car package based on type II Wald *χ*^2^ statistic [92]. Tukey’s multiple comparison test was applied for pairwise species and pairwise population comparisons using the *lsmeans* function of R emmeans package [93].

### 4.3. Environmental Heterogeneity

A Pearson’s correlation coefficient threshold of |0.8| between environmental variables (Supplementary Methods) was used to calculate variance inflation factor (VIF) separately for variables within each environmental category (bioclimate, ecology, and topography) (Supplementary Methods) using the *vifcor* function of R package usdm [94]. VIF values greater than 5 within each environmental category were removed. Pearson’s correlation coefficients of pairwise comparisons between variables were calculated and depicted in Figure S5. Environmental differences among species and among sample sites were assessed using PERMANOVA implemented in the *adonis* function of R package vegan [95], and pairwise comparisons assessed using the *pairwise.perm.manova* function of R package RVAideMemoire [96] with 999 permutations and a 5% false discovery rate (FDR).

### 4.4. AFLP Genetic Clustering and Relationships

Genetic homogeneous groups of individuals were assessed using sNMF algorithm [29] and DAPC [30] (Supplementary Methods). Individual assignments with *K* = 1–9 based on least-squares optimization using the *snmf* function of R LEA package [29]. The *find.clusters* and *dapc* functions of R adegenet package [31] were used in DAPC analysis. Genetic relationships among individuals were assessed using NJ and NN trees. The NJ tree was generated based on Nei’s genetic distances [33] using the *nei.dist* functions of R poppr package and the *nj* function of R package ape [97]. The NN tree was generated using the *neighborNet* function of R package phangorn [98]. The bootstrap values were calculated using 1000 bootstrap replicates with the *aboot* function of R package poppr for both NJ and NN trees.

### 4.5. Test for AFLP and MSAP F_ST_ Outliers

BAYESCAN and DFDIST were used to identify *F*_ST_ outliers (Supplementary Methods). BAYESCAN v.2.1 [34] was used to estimate the ratio of posterior probabilities of selection over neutrality (the posterior odds (PO)). A logarithmic scale of log_10_PO > 0.5 was defined as substantial evidence for selection over neutrality in BAYESCAN [99,100] (Supplementary Methods). DFDIST was used to estimate a distribution of observed *F*_ST_ versus *uH*_E_, and loci falling above the 95% confidence level of simulated distribution were identified as potential *F*_ST_ outliers. Global and pairwise population comparisons were performed in BAYESCAN and DFDIST.

### 4.6. AFLP Genetic Differentiation

AMOVA was used to estimate the hierarchical level of genetic differentiation using the *poppr.amova* function of R package poppr, and significance was tested using the *randtest* function of R package ade4 [101] with 9999 permutations. Both the total and outlier AFLP data were used in AMOVA. Among population, *F*_ST_ was also estimated using AFLP-SURV. Pairwise population *F*_ST_ was computed using ARLEQUIN based on the total and outlier AFLP data, and significance was tested with 10,000 permutations.

### 4.7. Associations of Genetic and Epigenetic Loci with Environmental Variables

LFMM [36] and Samβada [37] were employed to assess the associations of all genetic and epigenetic loci with environmental variables (Supplementary Methods). In LFMM, the number of latent factors was set to 3 and Z-scores of ten independent runs were combined using Fisher–Stouffer method [102]. *p* values were adjusted using the genomic inflation factor (*λ*) and a 1% FDR. Samβada was used to evaluate the associations of allele frequencies with the values of environmental variables. Both Wald and G scores with a 1% FDR for *p* value adjustment were used in assessing the fit of model with environmental variables against null model without environmental variables.

Loci found to be associated strongly with environmental variables assessed using Samβada and LFMM were further tested with a Bayesian logistic regression analysis implemented in the *brm* function of R brms package [38,39] (Supplementary Methods). Student’s *t* distribution with mean zero and seven degrees of freedom were used as the weakly informative priors, and the scale of the prior distribution was 2.5 for intercept and predictors using the *set_prior* function. Credible intervals (95%) were determined using the *posterior_summary* function.

### 4.8. AFLP and MSAP Isolation-by-Environment and Isolation-by-Distance

The correlations of the total and outlier Euclidean distance matrices with the Euclidean distance matrix of environmental variables were analyzed in a Mantel test using the *mantel* function of R vegan package with 999 permutations. Partial Mantel test was performed, controlling for geographic effect (latitude and longitude) using the *mantel.partial* function of R vegan package. MMRR was performed using the *MMRR* function [27] of R. Regression coefficients of IBE or adaptive divergence (*β*_E_) and IBD (*β*_D_) were obtained and significance was determined after 999 permutations. Models for redundancy analysis generated using the *rda* function of R vegan package were used in the forward selection [40] to test for the most important environmental variables explaining outlier genetic and epigenetic variations by using the *forward.sel* function of R adespatial package [103].

## 5. Conclusions

Understanding the roles that geography and environment play in speciation is an important issue in evolutionary biology [1,3,27,28,60,61]. Evolutionary processes, including incomplete lineage sorting and historical migration, might have played important roles in causing an *intermingling* of the *genealogical* relationships, revealed particularly in the NJ and NN trees, among individuals of the four closely related species in the *R. pseudochrysanthum* complex. A single ancestral phyletic line may diverge into a series of lineages, albeit with a shallow split in individual-based phylogenetic NJ and NN trees, adapting to rather different habitats. Our sampling of populations of the R. pseudochrysanthum complex, distributed at elevations below 1000 m and above 2000 m, and up to 3500 m, spanning a wide range of annual mean temperatures (5.1–18.9 °C), NDVI indexes (0.588–0.837), and PET indexes (953.2–1227.5), contributed to outlier genetic and epigenetic variations. The relatively stronger strength of IBE than IBD suggests spatial genetic and epigenetic structures driven by environmental conditions, and a strong IBE might play critical roles in causing the reproductive isolation and ecological speciation of closely related species in the *R. pseudochrysanthum* complex. However, locally adapted ecological lineages may risk extinction when encountering other environmental stressors during the course of migration. *R. hyperythrum* that grows in alpine tundra at high elevation may be vulnerable due to the detrimental effect of high-elevation environments on the growth of this species. Additionally, the introgression of adaptive genetic and epigenetic alleles and/or their combinations [82,104], harbored in low-elevation environments, into the genetic and epigenetic backgrounds of high-elevation locales, could be important, in particular, in the assisted migration program in the face of global change [105,106].

## Figures and Tables

**Table 1:** Site properties and genetic parameters of sampled populations of the *Rhododendron pseudochrysanthum* complex estimated based on the total AFLP variation.

Species Population	Longitude Latitude	Altitude (m)	*N*	%*p*	*uH*_E_ (SE)	*I*_A_ (*p*)	*r*_D_ (*p*)
*R. hyperythrum*							
Nanhutashan (HNHTS)	121.4381 24.3575	3500	41 (45)	63.5	0.2074 (0.009)	2.037 (0.001)	0.008 (0.001)
*R. morii*							
Alishan (MALS)	120.8006 23.51111	2100	18	65.1	0.2160 (0.009)	3.926 (0.001)	0.017 (0.001)
Hohuanshan (MHHS)	121.2575 24.11944	2800	14 (15)	57.3	0.2152 (0.009)	2.172 (0.001)	0.010 (0.001)
Tahsueshan (MTHS)	121.1281 24.31861	3085	8 (9)	60.4	0.2260 (0.010)	2.969 (0.001)	0.016 (0.001)
*R. pseudochrysanthum*							
Lulinshan (PLLS)	120.8719 23.46139	2862	20	69.8	0.2393 (0.009)	2.539 (0.001)	0.009 (0.001)
Hohuanshan (PHHS)	121.2619 24.13417	3400	17 (20)	64.3	0.2218 (0.009)	3.601 (0.001)	0.151 (0.001)
Tahsueshan (PTHS)	121.1303 24.32361	3121	19 (20)	56.0	0.2038 (0.010)	2.024 (0.001)	0.009 (0.001)
*R. rubropunctatum*							
Tsaigongken (RTGK)	121.5217 25.18972	886	13	59.4	0.2439 (0.010)	4.881 (0.001)	0.020 (0.001)
Tsankuangliao (RTKL)	121.8633 25.09444	630	21 (23)	62.5	0.2161 (0.010)	3.384 (0.001)	0.015 (0.001)
Total			171 (132)				
Average			17 (22)	62.03 (4.26)	0.2211 (0.009)		

*N*, Number of samples used in AFLP and MSAP (number in parenthesis); *%p*, the percentage of polymorphic loci; *uH*_E,_ unbiased expected heterozygosity. *I*_A_, index of association; *r*_D_, modified index of association.

**Table 2:** Genetic differentiation between species, between populations within species, and between nine populations of the *Rhododendron pseudochrysanthum* complex based on the total and outlier AFLP variation using analysis of molecular variance (AMOVA).

Source of Variation	Degree of Freedom	Sum of Squares	Percent Variation	*Φ* Statistics (*p*)
**Total Data**				
Between species	3	692.09	5.20	*Φ*_CT_ = 0.0520 (0.012)
Between populations within species	5	516.28	9.48	*Φ*_SC_ = 0.1000 (0.001)
Within populations	162	6083.74	85.32	*Φ*_ST_ = 0.1468 (0.001)
Total	170	7292.12	100	
**Outlier Data**				
Between species	3	178.32	22.83	*Φ*_CT_ = 0.2283(0.011)
Between populations within species	5	81.32	23.91	*Φ*_SC_ = 0.3099 (0.001)
Within populations	162	326.50	53.25	*Φ*_ST_ = 0.4675(0.001)
Total	170	586.140	100	

**Table 3:** Relative contribution (adjusted *R*^2^) and *F* test of environmental variables explaining outlier genetic and epigenetic variations of the *Rhododendron pseudochrysanthum* complex using a forward selection procedure.

Outlier Genetic/Epigenetic Variation	Category of Environmental Variables	Adjusted *R*^2^	Cumulative Adjusted *R*^2^	*F* Value (*p*)
**AFLP**				
	Bioclimate			
	BIO1	0.1576	0.1576	32.81 (0.001)
	BIO2	0.1070	0.2646	25.59 (0.001)
	BIO12	0.0429	0.3076	11.42 (0.001)
	Topology			
	Elevation	0.1394	0.1394	28.54 (0.001)
	Aspect	0.0518	0.1912	11.83 (0.001)
	Slope	0.0431	0.2343	10.45 (0.001)
	Ecology			
	PET	0.1150	0.1150	23.09 (0.001)
	CLO	0.0848	0.1998	18.90 (0.001)
	NDVI	0.0497	0.2495	12.13 (0.001)
	RH	0.0458	0.2953	11.85 (0.001)
	WSmean	0.0245	0.3197	6.97 (0.001)
**MSAP-m**				
	Bioclimate			
	BIO1	0.1925	0.1925	32.22 (0.001)
	BIO2	0.0382	0.2307	7.46 (0.001)
	Topology			
	Elevation	0.1272	0.1272	20.09 (0.001)
	Aspect	0.0336	0.1608	6.21 (0.001)
	Slope	0.0200	0.1804	4.08 (0.001)
	Ecology			
	PET	0.1664	0.1664	27.16 (0.001)
	NDVI	0.0350	0.2014	6.70 (0.001)
**MSAP-u**				
	Bioclimate			
	BIO1	0.3446	0.3446	69.89 (0.001)
	BIO2	0.1720	0.5166	47.25 (0.001)
	BIO12	0.0236	0.5402	7.63(0.001)
	Topology			
	Elevation	0.1772	0.1772	29.22 (0.001)
	Aspect	0.0613	0.2386	11.47 (0.001)
	Slope	0.0463	0.2848	9.35 (0.001)
	Ecology			
	NDVI	0.3518	0.3518	72.09 (0.001)
	PET	0.0996	0.4514	24.69 (0.001)

Aspect (0–360°) and slope (0–90°). BIO1, annual mean temperature; BIO2, mean of the difference of the monthly maximum and minimum temperatures; BIO12, annual precipitation; CLO, cloud cover; NDVI, normalized difference vegetation index; RH, relative humidity; PET, annual total potential evapotranspiration; WSmean, mean wind speed.

**Table 4:** Isolation-by-environment and isolation-by-distance tested using Mantel test, partial Mantel tests, and multiple matrix regression with randomization (MMRR). Euclidean distance matrices were generated based on AFLP, MSAP-m, and MSAP-u (G), geography (D), and environment (E). MMRR was used to infer the effects of geographic (*β*_D_) and environmental (*β*_E_) distances on genetic (AFLP) and epigenetic (MSAP-m and MSAP-u) distances. *R*^2^ represents the total amount of variation explained by both geographic and environmental factors. When outlier datasets were used, strong adaptive divergence can be inferred if significance was found controlling for geographic effect.

	Mantel Test Mantel *r* (*p*)			Partial Mantel Test Mantel *r* (*p*)		
	**G vs. E**	**G vs. D**	**E vs. D**	**G vs. E|D**		
**Total Data**						
AFLP	0.3634 (0.001)	0.4070 (0.001)	0.7175 (0.001)	0.1123 (0.001)		
MSAP-m	0.0300 (0.256)	−0.003 (0.481)	0.8167 (0.001)	0.0560 (0.062)		
MSAP-u	0.2844 (0.001)	0.2658 (0.001)	0.8167 (0.001)	0.1210 (0.001)		
**Outlier Data**						
AFLP	0.5286 (0.001)	0.4959 (0.001)		0.2858 (0.001)		
MSAP-m	0.3306 (0.001)	0.3003 (0.001)		0.1551 (0.001)		
MSAP-u	0.2545 (0.001)	0.2553 (0.001)		0.0825 (0.002)		
	**MMRR**					
	**G vs. E**	**G vs. D**	**E vs. D**	**G vs. E|D**		
				***R*** **^2^**	***β*** **_D_** **(*p*)**	***β*** **_E_** **(*p*)**
**Total Data**						
AFLP	0.2800 (0.001)	0.3014 (0.001)	0.6443 (0.001)	0.1902	0.2068 (0.001)	0.1467 (0.001)
MSAP-m	−0.0230 (0.664)	−0.0400 (0.435)	0.9431 (0.001)	0.0046	−0.1643 (0.001)	0.1319 (0.010)
MSAP-u	0.1925 (0.001)	0.1653 (0.001)	0.9431 (0.001)	0.0509	−0.1467 (0.001)	0.3307 (0.001)
**Outlier Data**						
AFLP	0.4678 (0.001)	0.4469 (0.001)		0.2897	0.2488 (0.001)	0.3074 (0.001)
MSAP-m	0.3247 (0.001)	0.3024 (0.001)		0.1074	−0.0175 (0.576)	0.3412 (0.001)
MSAP-u	0.2158 (0.001)	0.1885 (0.001)		0.0493	−0.1351 (0.005)	0.3431 (0.001)

## Data Availability

The data presented in this study are available on request from the corresponding author.

## Supplementary Materials

The following supporting information can be downloaded at: https://www.mdpi.com/article/10.3390/plants11091226/s1, Supplementary Methods; Table S1: Primer combinations, number of markers, and error rate per locus in AFLP and MSAP techniques for investigation in the *Rhododendron pseudochrysanthum* species complex; Table S2: Summary of Tukey’s post-hoc pairwise population comparisons of the mean unbiased expected heterozygosity (*uH*_E_) per locus using a linear mixed effect model. In linear mixed effect model, population was treated as a fixed factor and locus as a random factor based on the total AFLP variation of the *Rhododendron pseudochrysanthum* complex; Table S3: The 11 retained site environmental variables of the nine populations of the *Rhododendron pseudochrysanthum* species complex. See Table 1 for abbreviations of the nine populations; Table S4: *p* values of pairwise population comparisons of the 11 retained environmental variables of sample site of the *Rhododendron pseudochrysanthum* complex using PERMANOVA; Table S5: Pairwise *F*_ST_ (below diagonal) and *p* values (above diagonal) between populations of the *Rhododendron pseudochrysanthum* complex based on the total and outlier AFLP data using ARLEQUIN with 10,000 permutations; Table S6: Potential genetic (AFLP) and epigenetic (MSAP-m and MSAP-u) *F*_ST_ outliers identified by BAYESCAN and DFDIST associated with environmental variables assessed using Samβada, LFMM, and the *brm* function of R brms package; Table S7: Primer combination and amplified length for the 35 outliers presented in the Table S6. See Table S1 for E00, M00, and HM00 primer sequences. Figure S1: Evaluation of clustering scenarios based on (**a**) minimum cross-entropy and (**b**) Bayesian information criterion analyzed using LEA and DAPC, respectively; Figure S2: The neighbor-net tree of 171 individuals of four closely related species in the *Rhododendron pseudochrysanthum* complex. Tip labels for individuals of the four closely related species are colored: *R. hyperythrum* (blue), *R. morii* (violet red), *R. pseudochrysanthum* (dark green), and *R. rubropunctatum* (brown); Figure S3: Distribution of allele frequencies of the thirty-five *F*_ST_ outliers associated strongly with environmental variables across the nine populations of the *Rhododendron pseudochrysanthum* complex; Figure S4: The relationships of population pairwise *F*_ST_ with Euclidean distances between sample sites. Population pairwise *F*_ST_ was calculated using ARLEQUIN and Euclidean distances between sample sites were calculated based on geographic coordinates using the *dist* function of R stats package. Significance of the relationships were tested using Spearman’s correlation test (the *cor.test* function of R stats package) based on the total (blue line, *ρ* = 0.669, S = 2570, *p* < 0.0001) and outlier (red line, *ρ* = 0.634, S = 2844, *p* < 0.0001) AFLP datasets. The light blue and light red shades represent 95% confidence intervals of predicted values of simple linear regression; Figure S5: Pearson’s correlation coefficients between the 11 retained environmental variables. Aspect (0–360°) and slope (0–90°). BIO1, annual mean temperature; BIO2, mean of the difference of the monthly maximum and minimum temperatures; BIO12, annual precipitation; CLO, cloud cover; NDVI, normalized difference vegetation index, PET, annual total potential evapotranspiration; RH, relative humidity; WSmean, mean wind speed. References [107,108,109,110,111,112,113,114,115,116,117,118,119,120] are cite d in the supplementary materials.
