# Supplementary material for: Adaptive Divergence without Distinct Species Relationships Indicate Early Stage Ecological Speciation in Species of the Rhododendron pseudochrysanthum Complex Endemic to Taiwan
Source: Plants (Basel). 2022 Apr 30;11(9):1226. doi: 10.3390/plants11091226 (PMC9101530; doi:10.3390/plants11091226)
Supplement: Supplementary file 1 [file plants-11-01226-s001.zip › plants-1678461-supplementary.pdf]

Article

Adaptive Divergence without Distinct Species Relationships Indicate Early Stage Ecological Speciation in Species of the *Rhododendron pseudochrysanthum* Complex Endemic to Taiwan

Jia-Jia Cao [1,†], Yi-Shao Li [2,†], Chung-Te Chang [3], Jeng-Der Chung [4] and Shih-Ying Hwang [2,*]

1 Department of Life Science, National Taiwan University, 1 Roosevelt Road, Section 4, Taipei 10617, Taiwan; r05b21034@ntu.edu.tw
2 School of Life Science, National Taiwan Normal University, 88 Tingchow Road, Section 4, Taipei 11677, Taiwan; yishaoli@ntnu.edu.tw
3 Department of Life Science, Tunghai University, 1727 Taiwan Boulevard, Section 4, Taichung 40704, Taiwan; changchuante@gmail.com
4 Division of Silviculture, Taiwan Forestry Research Institute, 53 Nanhai Road, Taipei 10066, Taiwan; chung@tfri.gov.tw
* Correspondence: hsy9347@ntnu.edu.tw; Tel: +886-2-7749-6250
† These authors contributed equally to this work.

1. Supplementary Methods

1.1. DNA Extraction and Genotyping and Epigenotyping Error Rate

Silica gel dried, ground-up leaf powder was used in total DNA extraction based on a cetyltrimethyl ammonium bromide (CTAB) procedure [1]. Ethanol precipitated DNA was washed with 70% ethanol and dissolved in 200 μl TE buffer (pH 8.0). DNA concentration was quantified using a NanoDrop spectrophotometer (NanoDrop Technology, Wilmington, DE, USA). Error rate per locus of each AFLP primer combination was calculated based on amplification replicates obtained from three samples in each population. In MSAP, error rates of *Eco*RI-*Msp*I (*eMsp*I) and *Eco*RI-*Hpa*II (*eHpa*II), and a combined error rate (*eMsp*I + *eHpa*II – 2$e_{MspIeHpaII}$) for each primer combination were calculated [2] (Table S1). Loci with an error rate per locus greater than 5% were removed [3]. The mean error rate for AFLP was 3.76%. The mean error rate for *eMsp*I and *eHpa*II, respectively, were 1.75% and 1.85%, and the combined error rate for MSAP was 3.53%.

1.2. Environmental heterogeneity

Environmental variables of three categories: (1) 19 bioclimatic, (2) 3 topographic, and (3) 12 ecological variables were used in this study. Temperature and precipitation information of bioclimatic variables for sample sites at 30-sec spatial resolution (~ 1 km) were downloaded from the WorldClim 1.4 climate data [4]. Aspect, elevation, and slope (topographic variables) at 30-m resolution were obtained from Aster Global Digital Elevation Map (https://asterweb.jpl.nasa.gov/gdem.asp). Elevation is a topological factor [5] related to local climate of mountainous forest vegetation zones in Taiwan [6.7]. Twelve ecological variables included were normalized difference vegetation index (NDVI), enhanced vegetation index (EVI), leaf area index (LAI), fraction of absorbed photosynthetically active radiation (fPAR), relative humidity (RH), cloud cover (CLO), sunshine

hours (SunH), number of rainfall days per year (RainD), mean wind speed (WSmean), soil pH, annual total potential evapotranspiration (PET), and annual moisture index (MI).

Moderate resolution imaging spectroradiometer (MODIS) dataset MOD13A2 (1 km resolution) of NDVI and EVI, MOD15A2 dataset (500 m resolution) of LAI and fPAR, and MOD16A3 dataset (500 m resolution) of PET recorded during 2001–2013 were obtained from Land Process Distributed Active Archive Center (http://lpdaac.usgs.gov). We computed monthly mean values of these variables using a maximum-value composite procedure [8]. In addition, monthly mean values of RH, CLO, SunH, RainD, and WSmean were calculated with data obtained from the Data Bank for Atmospheric & Hydrologic Research (https://dbahr.pccu.edu.tw/, recorded in 1990–2013) at spatial resolution of 1 km using a universal spherical model of the Kriging method in ArcGIS [9]. We obtained soil pH values of the sample sites of this study based on the soil data of an island-wide 1,150 site investigation conducted in 1969–1986 by the Agriculture and Food Agency of Taiwan [10]. We used annual potential evapotranspiration derived from annual mean temperature and annual precipitation to calculate annual MI.

A correlation coefficient threshold of |0.8| between environmental variables was used to calculate the variance inflation factor (VIF) separately for each environmental category (bioclimate, ecology, and topology) using the *vifcor* function of R package usdm [11] in the R environment [12]. VIF values greater than 5 within each environmental category were removed. We retained eleven environmental variables: bioclimatic (BIO1, annual mean temperature; BIO2, mean of the difference of the monthly maximum and minimum temperatures; and BIO12, annual precipitation); topographic (aspect, elevation, and slope); and ecological (CLO, cloud cover; NDVI, normalized difference vegetation index; PET, annual total potential evapotranspiration; RH, relative humidity; and WSmean, mean wind speed) variables (Table S2). Pearson's correlation coefficients of pairwise comparisons between variables were calculated using the *cor* function of R stats package [12], and depicted in Supplementary Figure S5 with the *corrplot* function of the R corrplot package [13].

1.3. Genetic Clustering and Relationships Based on the Total AFLP Variation

Genetic homogeneous groups of individuals in the *R. pseudochrysanthum* complex were assessed using sNMF algorithm of landscape and ecological association (LEA) [14] and discriminant analysis of principal components (DAPC) [15], based on the total AFLP variation. In these analyses, individual assignments with *K* = 1–9 based on least-squares optimization using the *snmf* function of R LEA package [14]. The regularization parameter, iterations, and repetitions in *snmf* were set to 100, 200, and 10, respectively, in *snmf*, and other arguments set to defaults. The *find.clusters* and *dapc* functions of R adegenet package [16] were used in DAPC analysis. The mean minimal cross-entropy (CE) and the Bayesian information criterion (BIC) were, respectively, used to determine the best *K* in LEA and DAPC.

1.4. Test for AFLP and MSAP F_{ST} Outliers

BAYESCAN and DFDIST were used to identify F_{ST} outliers indicating signature of selection. BAYESCAN v.2.1 [17] implements a reversible-jump Markov chain Monte Carlo algorithm to estimate the ratio of posterior probabilities of selection over neutrality (the posterior odds (PO)). In

analysis using BAYESCAN, 200 pilot runs of 50,000 iterations were performed, followed by a sample size of 50,000 with a thinning interval of 20 among 10^6 iterations. Selection is detected when locus-specific component (α) is significantly different from zero. A positive α suggests divergent selection, while negative values suggest balancing or purifying selection. We used a criterion of a logarithmic scale of $\log_{10}$ (PO) > 0.5 as substantial evidence (q value < 0.05), corresponds to posterior probabilities between 0.76 and 0.91 and Bayes factors between 3 and 10 [18,19], for selection over neutrality for a locus under directional selection when $\alpha > 0$. DFDIST uses a modified Beaumont and Nichols model for dominant marker [20] to estimate a distribution of observed F_{ST} [21] versus uH_E [22]. AFLP and MSAP loci under selection were identified by comparing to a simulated neutral distribution. Parameters include critical frequency = 0.99; Zhivotovsky parameters = 0.25; trimmed mean F_{ST} = 0.3 (excluding 30% of highest and 30% of lowest F_{ST} values); smoothing proportion = 0.04; 500,000 resamplings; critical P = 0.05, and an average level of differentiation estimated were used to test for F_{ST} outliers in DFDIST. Loci falling above the 95% confidence level of simulated distribution were identified as potential F_{ST} outliers under directional selection using DFDIST. Global and pairwise population comparisons were performed in BAYESCAN and DFDIST.

1.5. Test for Genetic and Epigenetic Loci Associated with Environmental Variables

Latent factor mixed model (LFMM) [23] and Samβada [24] were employed to assess the associations of all genetic and epigenetic loci with environmental variables. LFMM considers the background level of population structure due to demographic history and IBD pattern, and a latent random factor was incorporated in a hierarchical Bayesian mixed effect model. Matrices of genetic and epigenetic variations were used as fixed factors. The number of latent factors was set to 3 considering the results obtained from DAPC analysis (Figure 2B). For each predictor, we performed ten LFMM runs with 10,000 iterations of the Gibbs sampling algorithm and a burn-in period of 5,000 cycles. Z-scores of ten independent runs were combined using Fisher-Stouffer method [25], and P values adjusted using the genomic inflation factor (λ). Additionally, an FDR correction of 1% was further used in P value adjustment using the *qvalue* function of R qvalue package [26]. Samβada uses a multiple univariate logistic regression approach to assess significant correlations of allele frequencies with the values of environmental variables. Both Wald and G scores with a 1% FDR for P value adjustment were used in assessing the fit of model with environmental variables against null model without environmental variables.

AFLP and MSAP loci detected by both BAYESCAN and DFDIST and found to be associated strongly with environmental variables assessed using Samβada and LFMM were further used in a Bayesian logistic regression analysis implemented in the *brm* function of R brms package [27,28]. Student's t distribution with mean zero and seven degrees of freedom were used as the weakly informative priors, and the scale of the prior distribution was 2.5 for intercept and predictors using the *set_prior* function of brms package. Four chains of *brm* Bernoulli regression models with logit link were performed with 2000 warm-up and 10,000 sampling steps. In *brm* analysis, convergence diagnostic found $\hat{R}$ very close to 1 and the bulk and effective sample size values representing overall sampling efficiencies for each predictor estimated were all greater than 10,000. The

posterior_summary function of brms package was employed to estimate 95% credible intervals for determination of significant correlations of F_{ST} outliers with environmental variables.

1.6. Map of Sampling Sites

The countries' boundary (polygon) map was derived from the default map database in ArcGIS v.10.3 [29]. The elevation gradients of Taiwan (background) were presented in ArcGIS based on the 20 m digital elevation model (DEM) [30]. The locations of the sampling sites were plotted using Tools in ArcGIS by their coordinates.

1.7. Logistic regression plot

Logistic regression was performed based on the generalized linear model with a logit link function and a binomial residual distribution. The presence/absence of the four loci (AC03_1652, AP04_3517, AP06_2592, and MP5_1240) were used as response variables and the environmental variables which strongly associated with the loci were used as predictors in analysis using the *glm* function of R. Individual logistic regression plot was visualized using the *visreg* function of R visreg package [31] and all plots were combined into one graph using the *ggplot2.multiplot* function of R easyGgplot2 package [32].

References

1. Doyle, J.J.; Doyle, J.L. A rapid DNA isolation procedure from small quantities of fresh leaf material. *Phytochem. Bull.* **1987,** *19,* 11–15.
2. Herrera, C.M.; Bazaga, P. Epigenetic differentiation and relationship to adaptive genetic divergence in discrete populations of the violet *Viola cazorlensis*. *New Phytol.* **2010,** *187,* 867–876.
3. Bonin, A.; Bellemain, E.; Bronken, E.P.; Pompanon, F.; Brochmann, C.; Taberlet, P. How to track and assess genotyping errors in population genetics studies. *Mol. Ecol.* **2004,** *13,* 3261–3273.
4. Hijmans, R.J.; Cameron, S.E.; Parra, J.L.; Jones, P.G.; Jarvis, A. Very high resolution interpolated climate surfaces for global land areas. *Int. J. Climatol.* **2005,** *25,* 1965–1978.
5. Amatulli, G.; Domisch, S.; Tuanmu, M.-N.; Parmentier, B.; Ranipeta, A.; Malczyk, J.; Jetz, W. A suite of global, cross-scale topographic variables for environmental and biodiversity modeling. *Sci. Data* **2018,** *5,* 180040.
6. Su, H.-J. Studies on the climate and vegetation types of the natural forests in Taiwan (II): Altitudinal vegetation zones in relation to temperature gradient. *Quart. J. Chinese Forest.* **1984,** *17,* 57–73 (1984). (in Chinese with English summary)
7. Li, C.-F., Chytrý, M., Zelený, D., Chen, M.-Y., Chen, T.-Y., Chiou, C.-R., Hsia, Y.-J., Liu, H.-Y.; Yang, S.-Z., Yeh, C.-L.; et al. Classification of Taiwan forest vegetation. *Appl. Veg. Sci.* **2013, *16,*** 698–719.
8. Huete, A.R.; Didan, K.; Miura, T.; Rodriguez, E.P.; Gao, X.; Ferreira, L.G. Overview of the radiometric and biophysical performance of the MODIS vegetation indices. *Remote Sens. Environ.* **2002,** *83,* 195–213.
9. Chang, C.-T.; Wang, S.-F.; Vadeboncoeur, M.A.; Lin, T.-C. Relating vegetation dynamics to temperature and precipitation at monthly and annual timescales in Taiwan using MODIS vegetation indices. *Int. J. Remote Sens.* **2014,** *35,* 598–620.
10. Chang, C.-T.; Lin, T.-C.; Lin, N.-H. Estimating the critical load and the environmental and economic impact of acid deposition in Taiwan. *J. Geogr. Sci.* **2009,** *56,* 39–58.
11. Naimi, B.; Hamm, N.A.S.; Groen, T.A.; Skidmore, A.K.; Toxopeus, A.G. Where is positional uncertainty a problem for species distribution modelling? *Ecography* **2014,** *37,* 191–203.
12. R Development Core Team. R: A Language and Environment for Statistical Computing. R Foundation for Statistical Computing Vienna, Austria, 2020. Available online: https://www.gbif.org/tool/81287/r-a-language-and-environment-for-statistical-computing.
13. Wei, T.; Simko, V.R. package "corrplot": visualization of a correlation matrix (version 0.84). **2017,** Available online: https://github.com/taiyun/corrplot (2017). (accessed on 6 January 2019).
14. Frichot, E.; François, O. LEA: an R package for landscape and ecological association studies. *Methods Ecol. Evol.*

2015, *6,* 925–929.

15. Jombart, T.; Devillard, S.; Balloux, F. Discriminant analysis of principal components: a new method for the analysis of genetically structured populations. *BMC Genet.* **2010,** *11,* 94.
16. Jombart, T.; Ahmed, I. adegenet 1.3-1: new tools for the analysis of genome-wide SNP data. *Bioinformatics* **2011,** *27,* 3070–3071.
17. Foll, M.; Gaggiotti, O. A genome scan method to identify selected loci appropriate for both dominant and codominant markers: a Bayesian perspective. *Genetics* **2008,** *180,* 977–993.
18. Jeffreys, H. *Theory of Probability.* Oxford University Press: Oxford, UK, 1961; ISBN 9780198503682.
19. Foll, M. *Bayescan 2.1 user manual.* 2012, Available online: http://cmpg.unibe.ch/software/BBayeSca/files/BayeScan2.1_manual.pdf. (accessed 8 march 2014).
20. Beaumont, M.A.; Nichols, R.A. Evaluating loci for use in the genetic analysis of population structure. *Proc. R. Soc. Lond. B. Biol. Sci.* **1996,** *263,* 1619–1626.
21. Weir, B.S.; Cockerham, C.C. Estimating F-statistics for the analysis of population structure. *Evolution* **1984,** *38,* 1358–1370.
22. Zhivotovsky, L.A. Estimating population structure in diploids with multilocus dominant DNA markers. *Mol. Ecol.* **1999,** *8,* 907–913.
23. Frichot, E.; Schoville, S.D.; Bouchard, G.; François O. Testing for associations between loci and environmental gradients using latent factor mixed models. *Mol. Biol. Evol.* **2013,** *30,* 1687–1699.
24. Stucki, S.; Orozco-terWengel, P.; Forester, B.R.; Duruz, S.; Colli, L.; Masembe, C.; Negrini, R.; Landguth, E.; Jones, M.R.; NEXTGEN Consortium, et al. High performance computation of landscape genomic models integrating local indices of spatial association. *Mol. Ecol. Resour.* **2017,** *17,* 1072–1089.
25. Brown, M.B. Method for combining non-independent, one-sided tests of significance. *Biometrics* **1975,** *31,* 987–992.
26. Storey, J.D.; Bass, A.J.; Dabney, A.; Robinson, D. qvalue: Q-value estimation for false discovery rate control. R package version 2.14.1. **2019,** Available online: http://github.com/StoreyLab/qvalue. (accessed on January 8, 2019).
27. Bürkner, P.-C. brms: An R Package for Bayesian Multilevel Models Using Stan. *J. Stat. Softw.* **2017,** *80,* 1–27.
28. Bürkner, P.-C. Advanced Bayesian Multilevel Modeling with the R Package brms. *The R J.* **2018,** *10,* 395–411.
29. Menon, S. ArcGIS 10.3. **2014,** The next generation of GIS is here. Environmental Systems Research Institute, Inc., CA, USA. Available online: http://www.esri.com/software/arcgis.
30. Open Government Data Providing Organization in Taiwan. Available online: http://data.gov.tw/node/35430.
31. Breheny, P.; Burchett, W. Visualization of Regression Models Using visreg. 2017 *The R. J.* **2017,** 9, 56–71.
32. Kassambara, A. **2014**, easyGgplot2: Perform and customize easily a plot with ggplot2. R package version 1.0.0.9000. Available online: http://www.sthda.com.

Table S1. Primer combinations, number of markers, and error rate per locus in AFLP and MSAP techniques for investigation in the *Rhododendron pseudochrysanthum* species complex.

AFLP			MSAP						
**Primer Combination**	**Number of Markers**	**Error Rate (%)**	**Primer Combination**	**Number of Markers**	**Number of MSAP-m Markers**	**Number of MSAP-u Markers**	**Error Rate (%)**		**Combined Error Rate (%)**
							*eHpa*II	*eMsp*I	
1 E00 CTA + M00 CTAT	44	3.367	1 E00 CAA + HM00 ACT	63	63	34	2.15	1.86	3.93
2 E00 CTA + M00 CTAC	56	3.232	2 E00 CCC + HM00 ATC	37	37	18	1.34	1.68	2.97
3 E00 CTA + M00 CTGA	32	5.051	3 E00 CCC + HM00 TGA	54	54	25	1.64	1.52	3.11
4 E00 CTA + M00 CTTC	25	5.556	4 E00 CGG + HM00 ACT	70	70	32	1.83	1.67	3.44
5 E00 CTA + M00 CTGT	49	2.669	5 E00 ATA + HM00 CAG	57	57	25	1.78	1.78	3.50
6 E00 TGA + M00 GAT	53	4.352	6 E00 ATA + HM00 GCG	76	76	41	2.01	1.43	3.38
7 E00 TGA + M00 CTGA	68	2.500	7 E00 ATA + HM00 GAA	58	58	31	1.86	1.74	3.54
8 E00 TGA + M00 CTTC	57	3.333	8 E00 TGA + HM00 CTA	58	57	28	1.93	2.37	4.21
			9 E00 TGT + HM00 CAG	59	59	23	1.88	1.48	3.30
			10 E00 TGT + HM00 CTA	49	49	17	2.03	1.94	3.89
Total (Average)	384 (48)	(3.758)		581 (58.1)	580 (58)	274 (27.4)	(1.85)	(1.75)	(3.53)

E00 (5′-GACTGCGTACCAATTC-3′), M00 (5′-GATGAGTCCTGAGTAA-3′), HM00 (*Hpa*II-*Msp*I, 5′-ATCATGAGTCCTGCTCGG-3′).

Table S2. Summary of Tukey's post-hoc pairwise population comparisons of the mean unbiased expected heterozygosity (uH_E) per locus using a linear mixed effect model. In linear mixed effect model, population was treated as a fixed factor and locus as a random factor based on the total AFLP variation of the *Rhododendron pseudochrysanthum* complex.

Comparison	Estimate	SE	df	*t*	*P* value
Species pair					
RH-RM	–0.00163	0.00946	3432	–0.172	0.9982
RH-RP	–0.01387	0.00946	3432	–1.466	0.4585
RH-RR	–0.00292	0.01004	3432	–0.291	0.9914
RM-RP	–0.01224	0.00669	3432	–1.83	0.2595
RM-RR	–0.0013	0.00748	3432	–0.173	0.9982
RP-RR	0.01095	0.00748	3432	1.463	0.46
Population pair					
HNHTS-MALS	0.005221	0.0116	3427	0.451	1
HNHTS-MHHS	–0.0017	0.0116	3427	–0.147	1
HNHTS-MTHS	–0.0084	0.0116	3427	–0.726	0.9984
HNHTS-PHHS	–0.02356	0.0116	3427	–2.036	0.5182
HNHTS-PLLS	0.012017	0.0116	3427	1.039	0.9821
HNHTS-PTHS	–0.03007	0.0116	3427	–2.599	0.1872
HNHTS-RTGK	–0.00137	0.0116	3427	–0.118	1
HNHTS-RTKL	–0.00448	0.0116	3427	–0.387	1
MALS-MHHS	–0.00692	0.0116	3427	–0.598	0.9996
MALS-MTHS	–0.01362	0.0116	3427	–1.177	0.9613
MALS-PHHS	–0.02878	0.0116	3427	–2.487	0.2385
MALS-PLLS	0.006796	0.0116	3427	0.587	0.9997
MALS-PTHS	–0.03529	0.0116	3427	–3.05	0.0585
MALS-RTGK	–0.00659	0.0116	3427	–0.569	0.9997
MALS-RTKL	–0.0097	0.0116	3427	–0.838	0.9957
MHHS-MTHS	–0.00671	0.0116	3427	–0.58	0.9997
MHHS-PHHS	–0.02186	0.0116	3427	–1.889	0.6213
MHHS-PLLS	0.013713	0.0116	3427	1.185	0.9598
MHHS-PTHS	–0.02838	0.0116	3427	–2.452	0.256
MHHS-RTGK	0.000328	0.0116	3427	0.028	1
MHHS-RTKL	–0.00278	0.0116	3427	–0.24	1
MTHS-PHHS	–0.01515	0.0116	3427	–1.31	0.9288
MTHS-PLLS	0.020421	0.0116	3427	1.765	0.7056
MTHS-PTHS	–0.02167	0.0116	3427	–1.873	0.6328
MTHS-RTGK	0.007036	0.0116	3427	0.608	0.9996
MTHS-RTKL	0.003926	0.0116	3427	0.339	1
PHHS-PLLS	0.035573	0.0116	3427	3.074	0.0545
PHHS-PTHS	–0.00652	0.0116	3427	–0.563	0.9998
PHHS-RTGK	0.022189	0.0116	3427	1.918	0.6015
PHHS-RTKL	0.019079	0.0116	3427	1.649	0.7775
**PLLS-PTHS**	**–0.04209**	**0.0116**	**3427**	**–3.637**	**0.0085**
PLLS-RTGK	–0.01339	0.0116	3427	–1.157	0.9652
PLLS-RTKL	–0.0165	0.0116	3427	–1.426	0.8882
PTHS-RTGK	0.028706	0.0116	3427	2.481	0.2416
PTHS-RTKL	0.025596	0.0116	3427	2.212	0.3979
RTGK-RTKL	–0.00311	0.0116	3427	–0.269	1

See Table 1 for population codes. Bold letter indicates significant pair comparison.

Table S3. The 11 retained site environmental variables of the nine populations of the *Rhododendron pseudochrysanthum* species complex. See **Table 1** for abbreviations of the nine populations.

Population	BIO1	BIO2	BIO12	Aspect	Elevation	Slope	CLO	NDVI	PET	RH	WSmean
HNHTS	5.1	6.9	3588	253	3500	29	6.9	0.588	953.2	78.3	2.58
MHHS	7.8	7.3	4043	259	2800	22	6.1	0.823	981	79.7	5.18
MTHS	6.8	7.2	3600	101	3085	41	6.2	0.811	1048.5	77.8	2.34
MALS	11.4	8.3	3814	306	2100	36	6.1	0.835	1050.1	85.9	1.23
PTHS	8.8	7.3	3191	30	3121	29	6.2	0.816	1048.5	77.8	2.35
PHHS	8.5	7.3	4006	284	3400	25	6.1	0.837	970.1	79.9	5.39
PLLS	9.4	7.9	3595	308	2862	19	6	0.785	1286.8	79.2	3.4
RTGK	16.8	5.9	3807	344	886	10	7.5	0.824	1189.9	88.5	3.04
RTKL	18.9	5.7	3586	242	630	19	7.5	0.779	1227.5	78.5	2.65

See Table 1 for population codes. Aspect (0–360°) and slope (0–90°). BIO1, annual mean temperature (°C); BIO2, mean of the difference of the monthly maximum and minimum temperatures (°C); BIO12, annual precipitation (mm); CLO, cloud cover (%); NDVI, normalized difference vegetation index (unitless), PET, annual total potential evapotranspiration (kg/m^2/year); RH, relative humidity (%); WSmean, mean wind speed (m/s).

Table S4. *P* values of pairwise population comparisons of the 11 retained environmental variables of sample site of the *Rhododendron pseudochrysanthum* complex using PERMANOVA.

	***P* value**								
	**HNHTS**	**MALS**	**MHHS**	**MTHS**	**PHHS**	**PLLS**	**PTHS**	**RTGK**	**RTKL**
MALS	1	-	-	-	-	-	-	-	
MHHS	1	0.0019	-	-	-	-	-	-	
MTHS	1	1	1	-	-	-	-	-	
PHHS	0.0019	0.0019	0.0019	0.0019	-	-	-	-	
PLLS	1	0.0019	0.0019	0.0019	0.0019	-	-	-	
PTHS	1	1	1	0.0019	1	0.0019	-	-	
RTGK	0.0019	1	0.0019	1	0.0019	0.0019	0.0019	-	
RTKL	0.0019	1	0.0019	1	1	1	0.0019	1	

See Table 1 for population codes. Significance determined by 999 permutations and a false discovery rate of 5%.

Table S5. Pairwise F_{ST} (below diagonal) and *P* values (above diagonal) between populations of the *Rhododendron pseudochrysanthum* complex based on the total and outlier AFLP data using ARLEQUIN with 10,000 permutations. .

	HNHTS	MALS	MHHS	MTHS	PHHS	PLLS	PTHS	RTGK	RTKL
**Total**									
HNHTS		0	0	0	0	0	0	0	0
MALS	0.12793		0	0.00098	0	0.00293	0	0	0
MHHS	0.08263	0.08553		0.02344	0	0	0	0	0
MTHS	0.0853	0.091	0.03865		0.04492	0	0	0	0
PHHS	0.11359	0.10143	0.06604	0.04404		0.00098	0	0	0
PLLS	0.15871	0.05195	0.11628	0.11931	0.06925		0	0	0
PTHS	0.1352	0.1886	0.15438	0.12088	0.08844	0.16418		0	0
RTGK	0.21428	0.15307	0.18018	0.12898	0.1293	0.13454	0.18921		0
RTKL	0.21388	0.19028	0.20002	0.15372	0.13749	0.17912	0.17751	0.09804	
**Outlier**									
HNHTS		0	0	0	0	0	0	0	0
MALS	0.53485		0	0	0	0	0	0	0
MHHS	0.29091	0.22818		0.25648	0	0	0	0	0
MTHS	0.21849	0.38059	0.02322		0	0	0	0	0
PHHS	0.4719	0.43906	0.33189	0.30561		0	0	0	0
PLLS	0.55189	0.29353	0.32069	0.39152	0.11987		0	0	0
PTHS	0.28728	0.5567	0.41549	0.34876	0.37306	0.48619		0	0
RTGK	0.57015	0.51567	0.39757	0.36866	0.38932	0.42503	0.42245		0
RTKL	0.61074	0.60036	0.51155	0.49878	0.48346	0.54103	0.47093	0.22368	0

See Table 1 for population codes.

Table S6. Potential genetic (AFLP) and epigenetic (MSAP-m and MSAP-u) F_{ST} outliers identified by BAYESCAN and DFDIST associated with environmental variables assessed using Samβada, LFMM, and the *brm* function of R brms package.

Outliers	Global/pair	BAYESCAN $\log_{10}$(PO)	Samβada[a], LFMM[b], brms[c]										
			Bioclimate			Topology			Ecology				
			BIO1	BIO2	BIO12	Aspect	Elevation	Slope	CLO	NDVI	PET	RH	WSmean
**AFLP**													
AC01_2349	Global	2.1581	c	a, b, c	a, b, c	a, c		c	a, c	a		c	
AC02_2897	Global	1.1734	c	a, b, c	b, c	b, c	c	c	c			a, b, c	
AC02_3094	Global	1.9717	a, b, c	a, b, c		c	a, b, c	a	a, b, c	c	a	c	
AC03_1652*	Global	4.0969	a, b, c		c	c	a, c	a, c		a, c	a, b, c	c	b
	MHHS vs. RTKL	1.2175	a, b				a	a		a	a, b		b
	MTHS vs. RTKL	1.1235	a, b				a	a		a	a, b		b
AC04_2218	Global	2.3507	a, c	c	a, b, c	a, c	c		c	a	a, b, c		b, c
AC05_1828	Global	0.56247	b	a, b, c	a, b, c	b		a, b, c	b, c				a, c
AC05_2733	Global	4.2218	a, b, c	c	c	c	c	a, c		a, b	a, b, c		a, b, c
AC05_3404	Global	0.69873	a, c	c	a, b, c	a, b, c	a, c				a, b, c	a, c	b
AP03_1836	Global	0.98165	a, b, c	a, b, c			a, b, c	a	a, b, c		a, c		
AP03_1964	Global	1.1651		a, b, c	a, c	c		c	a, c			c	
AP04_2327	Global	1.5567	a, b, c	a, b			a, b, c	a	a, c	a, c	a	a	
AP04_3517*	Global	1000	a, b, c				a, c	a, c		a, b	a, b, c	a, c	b, c
	HNHTS vs. RTGK	1.7922	a, b				a	a		a, b	a, b	a	b
	HNHTS vs. RTKL	2.1842	a, b				a	a		a, b	a, b	a	b
AP06_1525	Global	1.3091	a, c	c	a, b, c	a, b, c	a, c			a	a, b, c	a, b, c	b
AP06_2592*	Global	1000	a, b, c	a, b	c	c	a, b, c	a	a, b, c	c	a		c
	PHHS vs. RTGK	0.76459	a, b	a, b			a, b	a	a, b		a		
	PLLS vs. RTGK	1.0123	a, b	a, b			a, b	a	a, b		a		
AP06_2736	Global	1000	c	c			c	a, c	c	a, b	a, b, c	a, c	b, c
AP06_3422	Global	2.2494		a, b, c	c	c	c	c	a, b, c			c	
**MSAP_m**													
MP1_2426	Global	0.69059	b, c	c		a, b		b, c	b	a, b	a, c	c	
MP5_1240	Global	0.53581	b, c			a, b, c		b, c	a, b	a, b	a, b, c		
MP6_2133	Global	1.9134	b, c	c		a, c		b, c		a, b, c	c		
MP10_3312	Global	3.0651	b, c		c	a, c	a, b	b, c	b	b	c		

**MSAP_u**												
UP1_1044	Global	3.61968	c	c		a, b, c	a, b	b, c		a, b, c	a, b, c	a, b, c
UP1_1489	Global	4.699	c	c	c	a, b, c	a, b	b, c		a, b, c	a, b, c	a, b
UP1_1793	Global	1000	c	c		a, b, c	b, c	b, c		a, b, c	a, b, c	a, b, c
UP1_2391	Global	2.28	c	c		a, b, c	a, b	b, c		a, b, c	a, b, c	a, b
UP3_1006	Global	1000	c	c	c	a, b, c	a, b	b, c		a, b	b, c	a, b
UP5_1076	Global	1000	c	c		a, b, c	a, b, c	b, c		a, b	b, c	a, b, c
UP5_1855	Global	1000	c		c	a, b, c	a, b	b, c		a, b	a, b, c	a, b, c
UP5_1946	Global	1000	c		c	a, b, c	b, c	b, c		a, b	a, b, c	a, b, c
UP6_1385	Global	3.5527	c			a, b, c	a, b, c	b, c		a, b	b, c	a, b, c
UP7_1014	Global	4.3979	a, b, c	c	c	a, b	a, b, c	b		a, b, c	a, b, c	a, b, c
UP7_2306	Global	1000	c	c	b	a, b, c	b	c		a, c		b
UP9_1333	Global	1000	c	c	c	b, c	b, c	b, c		a, b	b, c	a, b, c
UP9_1383	Global	1000	c		c	a, b, c	a, b	b, c		a, b	a, b, c	a, b, c
UP9_3301	Global	1000	c			a, b, c	a, b, c	b, c		a, b	b, c	a, b, c
UP11_1499	Global	1000	c	c	c	a, b, c	a, b	b, c		a, b, c	a, b, c	a, b

Thirty-five potential outliers were identified by BAYESCAN and DFDIST associated with environmental variables analyzed with regression approaches including Samβada, LFMM, and brms. *, F_{ST} outliers detected by BAYESCAN and DFDIST in both global and pair population comparisons. a and b represent significant correlations of AFLP markers with individual environmental variables identified by Samβada and LFMM, respectively. c represents significance based on 95% posterior credible intervals for the potential outliers found to have strongly correlated with specific environmental variable(s) using the brm function of R package brms. Aspect (0–360°) and slope (0–90°). BIO1, annual mean temperature; BIO2, mean of the difference of the monthly maximum and minimum temperatures; BIO12, annual precipitation; CLO, cloud cover; NDVI, normalized difference vegetation index; RH, relative humidity; PET, annual total potential evapotranspiration; WSmean, mean wind speed.

Table S7. Primer combination and amplified length for the 35 outliers presented in the Table S6. See Table S1 for E00, M00, and HM00 primer sequences.

Outliers	Primer combination	Length (bp)
**AFLP**		
AC01_2349	E00 CTA + M00 CTAT	234.9
AC02_2897	E00 CTA + M00 CTAC	289.7
AC02_3094	E00 CTA + M00 CTAC	309.4
AC03_1652	E00 CTA + M00 CTGA	165.2
AC04_2218	E00 CTA + M00 CTTC	221.8
AC05_1828	E00 CTA + M00 CTGT	182.8
AC05_2733	E00 CTA + M00 CTGT	273.3
AC05_3404	E00 CTA + M00 CTGT	340.4
AP03_1836	E00 TGA + M00 GAT	183.6
AP03_1964	E00 TGA + M00 GAT	196.4
AP04_2327	E00 TGA + M00 CTGA	232.7
AP04_3517	E00 TGA + M00 CTGA	351.7
AP06_1525	E00 TGA + M00 CTTC	152.5
AP06_2592	E00 TGA + M00 CTTC	259.2
AP06_2736	E00 TGA + M00 CTTC	273.6
AP06_3422	E00 TGA + M00 CTTC	342.2
**MSAP_m**		
MP1_2426	E00 CAA + HM00 ACT	242.6
MP5_1240	E00 ATA + HM00 CAG	124.0
MP6_2133	E00 ATA + HM00 GCG	213.3
MP10_3312	E00 TGT + HM00 CAG	331.2
**MSAP_u**		
UP1_1044	E00 CAA + HM00 ACT	104.4
UP1_1489	E00 CAA + HM00 ACT	148.9
UP1_1793	E00 CAA + HM00 ACT	179.3
UP1_2391	E00 CAA + HM00 ACT	239.1
UP3_1006	E00 CCC + HM00 TGA	100.6
UP5_1076	E00 ATA + HM00 CAG	107.6
UP5_1855	E00 ATA + HM00 CAG	185.5
UP5_1946	E00 ATA + HM00 CAG	194.6
UP6_1385	E00 ATA + HM00 GCG	138.5
UP7_1014	E00 ATA + HM00 GAA	101.4
UP7_2306	E00 ATA + HM00 GAA	230.6
UP9_1333	E00 TGA + HM00 CTA	133.3
UP9_1383	E00 TGA + HM00 CTA	138.3
UP9_3301	E00 TGA + HM00 CTA	330.1
UP11_1499	E00 TGT + HM00 CTA	149.9

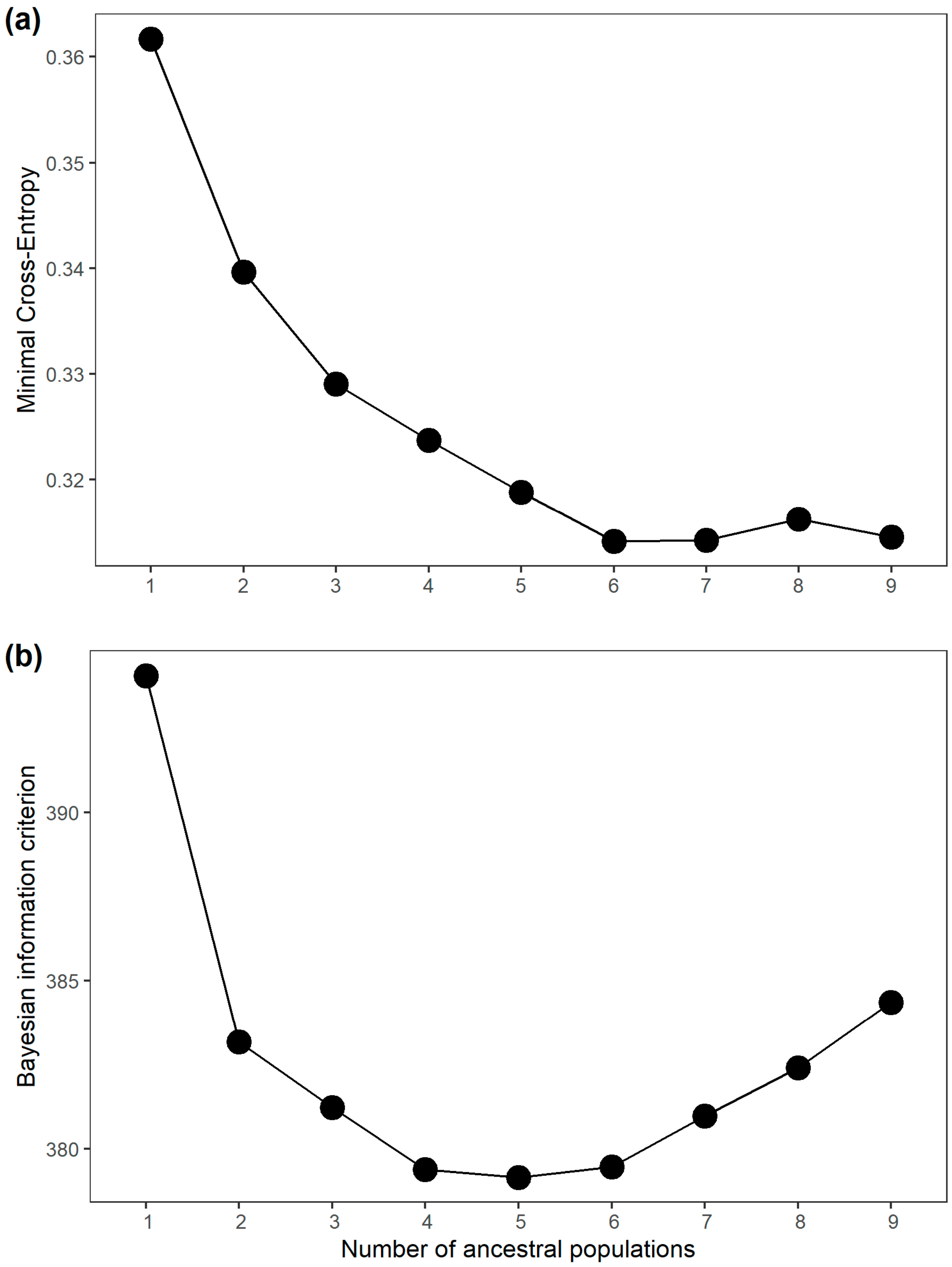

Figure S1. Evaluation of clustering scenarios based on **(a)** minimum cross-entropy and **(b)** Bayesian information criterion, respectively, analyzed using LEA and DAPC.

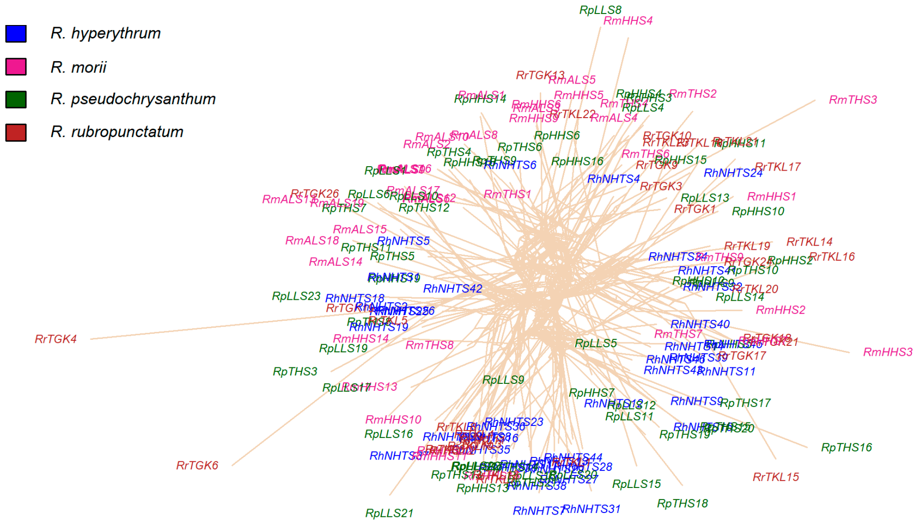

Figure S2. The neighbor-net tree of 171 individuals of four closely related species in the *Rhododendron pseudochrysanthum* complex. Tip labels for individuals of the four closely related species are colored: *R. hyperythrum* (blue), *R. morii* (violet red), *R. pseudochrysanthum* (dark green), and *R. rubropunctatum* (brown).

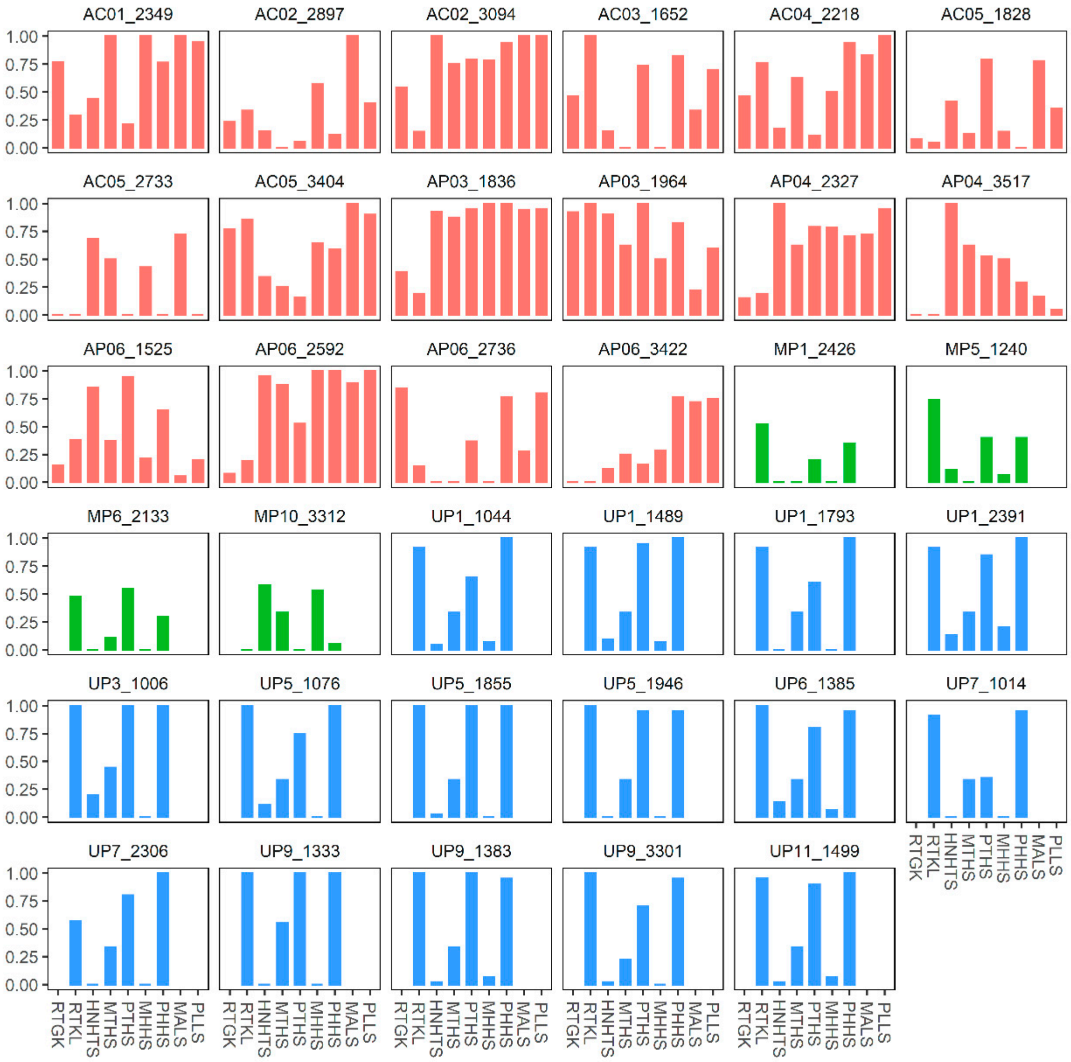

Figure S3. Distribution of allele frequencies of the thirty-five F_{ST} outliers associated strongly with environmental variables across the nine populations of the *Rhododendron pseudochrysanthum* complex.

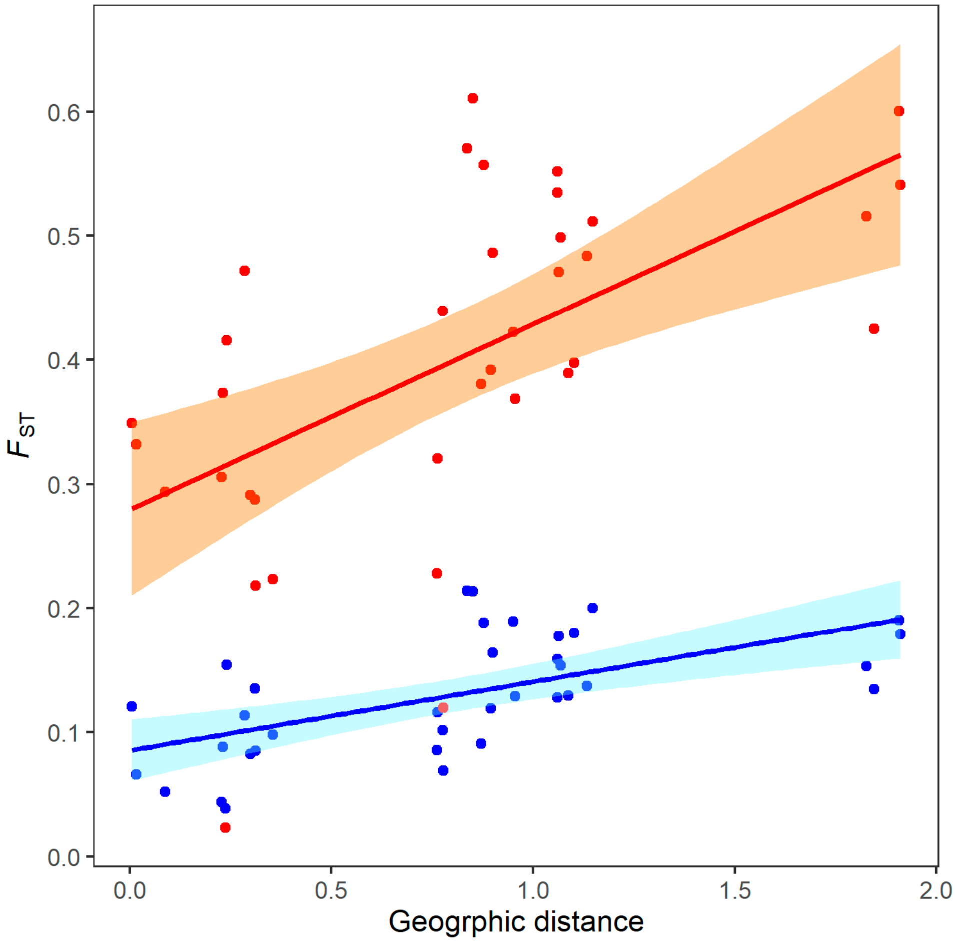

Figure S4. The relationships of population pairwise F_{ST} with Euclidean distances between sample sites. Population pairwise F_{ST} was calculated using ARLEQUIN and Euclidean distances between sample sites were calculated based on geographic coordinates using the *dist* function of R stats package. Significance of the relationships were tested using Spearman's correlation test (the *cor.test* function of R stats package) based on the total (blue line, $\rho = 0.669$, $S = 2570$, $P < 0.0001$) and outlier (red line, $\rho = 0.634$, $S = 2844$, $P < 0.0001$) AFLP datasets. The light blue and light red shades represent 95% confidence intervals of predicted values of simple linear regression.

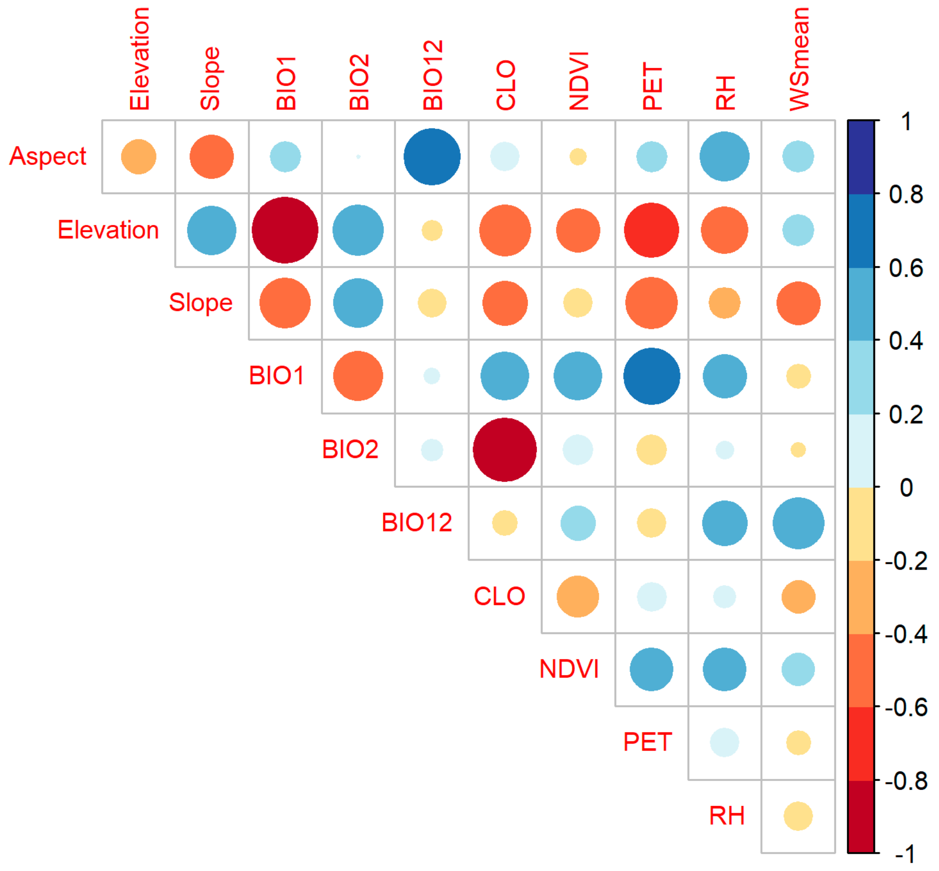

Figure S5. Pearson's correlation coefficients between the 11 retained environmental variables. Aspect (0–360°) and slope (0–90°). BIO1, annual mean temperature; BIO2, mean of the difference of the monthly maximum and minimum temperatures; BIO12, annual precipitation; CLO, cloud cover; NDVI, normalized difference vegetation index, PET, annual total potential evapotranspiration; RH, relative humidity; WSmean, mean wind speed.
